# NIR-I Dye-Based Probe: A New Window for Bimodal Tumor Theranostics

**DOI:** 10.3389/fchem.2022.859948

**Published:** 2022-03-23

**Authors:** Fan Zheng, Xueyan Huang, Jipeng Ding, Anyao Bi, Shifen Wang, Fei Chen, Wenbin Zeng

**Affiliations:** ^1^ Xiangya School of Pharmaceutical Sciences, Central South University, Changsha, China; ^2^ Hunan Key Laboratory of Diagnostic and Therapeutic Drug Research for Chronic Diseases, Changsha, China

**Keywords:** NIR-I fluorescence imaging, bimodal imaging, nanoparticles, cancer, theranostics

## Abstract

Near-infrared (NIR, 650–1700 nm) bioimaging has emerged as a powerful strategy in tumor diagnosis. In particular, NIR-I fluorescence imaging (650–950 nm) has drawn more attention, benefiting from the high quantum yield and good biocompatibility. Since their biomedical applications are slightly limited by their relatively low penetration depth, NIR-I fluorescence imaging probes have been under extensive development in recent years. This review summarizes the particular application of the NIR-I fluorescent dye-contained bimodal probes, with emphasis on related nanoprobes. These probes have enabled us to overcome the drawbacks of individual imaging modalities as well as achieve synergistic imaging. Meanwhile, the application of these NIR-I fluorescence-based bimodal probes for cancer theranostics is highlighted.

## 1 Introduction

Cancer is one of the major public health problems across the world (Torre et al., 2015; Feng et al., 2019). Advanced imaging techniques undoubtedly play an indispensable role in the development of diagnostic and therapeutic approaches for cancer. With the advantages of high sensitivity, high selectivity, noninvasiveness, and real-time visualization, fluorescence imaging (FI) has been widely used in the clinic to diagnose diseases and precisely guide resection in surgery (Jewell et al., 2014; Pramod Kumar et al., 2020). The working mechanism of realizing imaging functionality underscores the need for proper reporter groups as tracers. The reporter group includes a variety of fluorescent dyes, fluorescent proteins, upconversion nano-particles, quantum dots, and so on (Adhikari et al., 2016; Laviv et al., 2016; Kostiv et al., 2017; Saljoghi et al., 2020). They can absorb the energy of photons to reach an excited state, and then return to the ground state accompanied by the release of energy as fluorescence. Among them, fluorescent dyes are considered as ideal reporters due to their superiority of simple components, flexible structure, and good biocompatibility.

However, conventional visible fluorescence imaging always suffers from low tissue penetration depth in *in vivo* optical imaging, which is attributed to the fact that light with a short wavelength can be more easily scattered and absorbed by endogenous biological tissues including skin, fat, and blood (Smith et al., 2009; Sordillo et al., 2014). The emergence of near-infrared (NIR) fluorescence imaging has brought a new chance with the merits of deeper tissue penetration and minor auto-fluorescence interference, which can afford distinct signals with increased spatial resolution and imaging sensitivity to track target tissues. Generally, it can be further divided into first near-infrared (NIR-I, 650–950 nm) and second near-infrared (NIR-II, 1,000–1700 nm) fluorescence imaging (Qi et al., 2018; Kubicek-Sutherland et al., 2020; Moreno et al., 2020; Shinn et al., 2021). Although possessing a better signal-to-noise ratio and depth-to-resolution ratio, NIR-II fluorophores still show deficiencies in stability, quantum yield, and biocompatibility (Bhavane et al., 2018; Kenry et al., 2018; Qian et al., 2021; Kang et al., 2022). Hence, FI which we have mentioned in this review refers to NIR-I fluorescence imaging.

To make a further breakthrough in imaging depth and efficiency, the combinations of FI with other imaging modalities, such as fluorescence imaging/nuclear medicine imaging (FI/NMI), fluorescence imaging/magnetic resonance imaging (FI/MRI), fluorescence imaging/computed tomography (FI/CT), and fluorescence imaging/photoacoustic imaging (FI/PAI) could be intelligent solutions. Besides the improvement in penetration depth, different modalities have other different properties. The details of each modality are described in Table 1. For example, with extremely high sensitivity, NMI can offer quantitative information of the local probe (Boellaard et al., 2009). Based on the high spatial resolution, MRI can provide unmatched soft tissue details, while CT is capable of capturing images of anatomical information (Murakami and Nakahara, 2014; Porrino et al., 2022). Furthermore, given the quick and accurate acquisition process, PAI can provide images with high spatial–temporal resolution (Martel et al., 2014). However, radiation exposure is the main issue in NMI as well as in CT imaging, and MRI often suffers from its low temporal resolution. Moreover, MRI, CT, and PAI are all limited by their low sensitivity. Under these circumstances, the application of FI can make up for the deficiencies of these modalities, and furthermore, FI-based bimodal probes can achieve effective imaging in tumor theranostics with complementary advantages.

**TABLE 1:** Details of the mentioned modalities.

Modality	Working mechanism	Spatial resolution	Sensitivity (mol/L)	Depth of penetration	Acquisition time	Cost	Main characteristics	
							Advantages	Disadvantages
FI	To absorb the energy of photons to reach the excited state, and then return to the ground state accompanied by the release of energy as fluorescence	∼2 mm	High (10^−9^–10^−12^)	<1 cm	Sec/min	Low	High sensitivity	Low penetration depth
							High temporal resolution	
NMI	To detect the gamma rays directly or indirectly from the decay of radionuclides	∼7 mm	High (10^−10^–10^−12^)	Unlimited	Min	High	Providing functional information	Radiation
								Low spatial resolution
MRI	To record the spatial distribution of the water protons with different relaxation rates	∼1 mm	Low (10^−3^–10^−5^)	Unlimited	Min/hr	High	Providing soft tissue details	Low temporal resolution
								Low sensitivity
CT	To detect the X-rays attenuation degrees of specific tissues	∼0.5 mm	Very low	Unlimited	Min	Relatively high	Providing anatomical information	Radiation
								Low sensitivity
PAI	To collect the acoustic waves caused by the pulsed laser	∼0.2 mm	Relatively high	Deep up to 12 cm	Sec/min	Low	High spatial–temporal resolution	Relative low penetration depth

More than this, the development of multimodality imaging is further promoted *via* the blossoming of nanotechnology. On the one hand, characteristics of nanoparticles (NPs), including large surface-to-volume ratio, passive accumulation at tumor endothelial cells, high labeling capacity, and prolonged blood circulation, are advantageous in this field. On the other hand, nanomaterials make it easier to combine different functional blocks. Remarkably, numerous multimodal probes can be integrated within one single nanoparticle, which greatly improves the delivery ability for cancer diagnosis.

This review summarizes the advances of NIR-I fluorescent dye-related bimodal imaging of tumors in the recent 5 years extensively. We mainly focus on the rational design strategies and the representative paradigms of versatile probes, while the challenges and outlook have also been discussed. We hope that this review will raise extensive interest and offer suggestions for the development of biomedical multimodality imaging in the diagnosis of cancer.

## 2 Bimodal Imaging of Tumor Based on Near-Infrared-I Fluorescence

Tumor-specific imaging is achieved by distinguishing the distinction between malignant and healthy tissues. Tumor tissues comprise not only tumor cells but also the microenvironment where they exist. With this in mind, the design strategies of imaging probes can be developed based on the discrepancies between tumor and normal tissues, such as lower pH, hypoxia, and overexpression of enzymes and integrins (Imamura et al., 2018; Shim et al., 2019; Tansi et al., 2020).

Various multimodality imaging techniques, which refer to the combination of NIR-I fluorescence imaging with other imaging modalities, have been reported as advanced diagnostic approaches for cancer. Hopefully, designing dual-modal probes for FI/NMI, FI/MRI, FI/CT imaging, and FI/PAI systems will compensate for the weakness of individual modality and enable synergistic imaging.

### 2.1 Fluorescence Imaging/Nuclear Medicine Imaging

NMI has been commonly used in cancer diagnosis owing to its infinite penetration depth and high temporal resolution. There are two main modalities: single-photon emission computed tomography (SPECT) and positron emission tomography (PET). Since SPECT requires a collimator for imaging which is needless for PET, its detection sensitivity is usually lower than PET. Metallic or nonmetallic radionuclides are commonly used as tracers to generate images in NMI. The working mechanism is the detection of gamma (γ) rays directly or indirectly from the decay of these radionuclides. Tc-99m, Ga-67, I-123, I-125, and I-131 are often utilized as radioactive atoms in SPECT imaging (Beer et al., 1990; Al-Suqri and Al-Bulushi, 2015), while Ga-68, Cu-64, F-18, C-11, and I-124 are used in PET imaging (Nakajima et al., 2017). Hence, the commercialized NMI probes are usually radiolabeled molecules. For example, ^18^F-fluorodeoxyglucose (^18^F-FDG) has been widely applied as a PET radiotracer to evaluate neoplastic diseases.

Bimodal imaging techniques, such as PET-CT and PET-MRI, have already been well established for clinical use. These clinical NMI-based bimodal imaging technologies undoubtedly hold the potential for improved diagnostic evaluation by merging functional information with anatomical information or soft tissue details. However, their solutions to the high radiation exposure and related health risks remain limited. Meanwhile, difficulty in the diagnosis of tiny lesions in the early stages of cancer hinders their further biological application. Since FI has high sensitivity but with significantly lower toxicity, the bimodal FI/NMI probes enable a lower dose to offer a better image in the early diagnosis of tumors compared to individual NMI or other NMI-based bimodal probes (Wang X. et al., 2018). The combination of FI and NMI can overcome the obstacles of single modality imaging, allowing imaging with little radiation burden and unlimited penetration depth. For the construction of the bimodal probes in FI/NMI, however, the direct use of radiolabeled fluorescent dyes lack clear targeting sites (Kanagasundaram et al., 2021; Peng et al., 2021). In this context, modified molecular probes and passively accumulated nanoparticles have been attributed to implement specific tumor uptake.

#### 2.1.1 Fluorescence Imaging/Single-Photon Emission Computed Tomography Imaging

SPECT is used more frequently than PET in the clinic due to wider availability of its scanners and radionuclides. The core of any radiotracer in SPECT imaging is its gamma-emitting radionuclide. ^99m^Tc and ^111^In have been the most commonly used radionuclides in FI/SPECT dual-modal imaging. ^99m^Tc has been used for medical imaging in 80% of cases around the world, and its decay product has little effect on the image quality (Lee and Grp, 2019). Also, benefiting from its physical properties, ^99m^Tc (Eγ = 140.5 keV; t_1/2_ = 6.02 h) has minimal harmful radiation to the patients. Nevertheless, given the relatively short half-life of ^99m^Tc, ^111^In (t_1/2_ = 2.8 d) has gained much attention recently.

**FIGURE 1:**
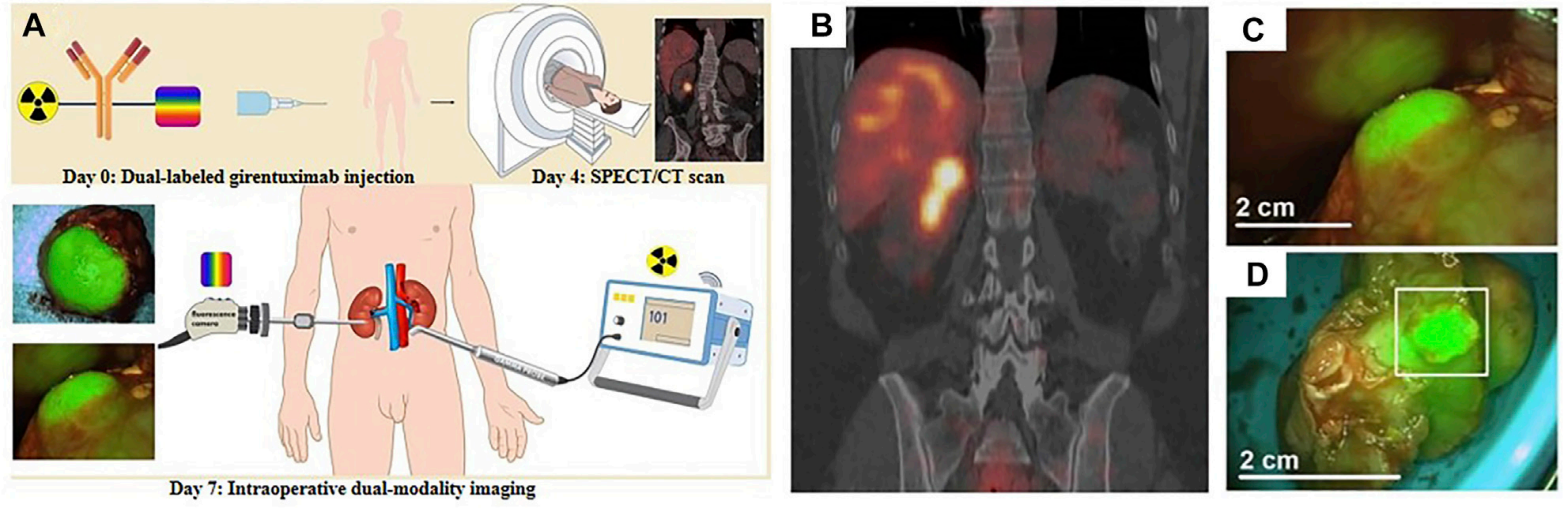
**(A)** Schematic illustration of ^111^In-DOTA-girentuximab-IRDye800CW in clinical applications. **(B)** Preoperative SPECT/CT imaging of patient #11 after injection of ^111^In-DOTA-girentuximab-IRDye800CW. **(C)** Intraoperative FI of patient #11. **(D)** Assessment of the resected tumor specimen with FI (patient #11). Reproduced with permission from Hekman et al. (2018). Copyright 2018, Ivyspring International Publisher.

**FIGURE 2:**
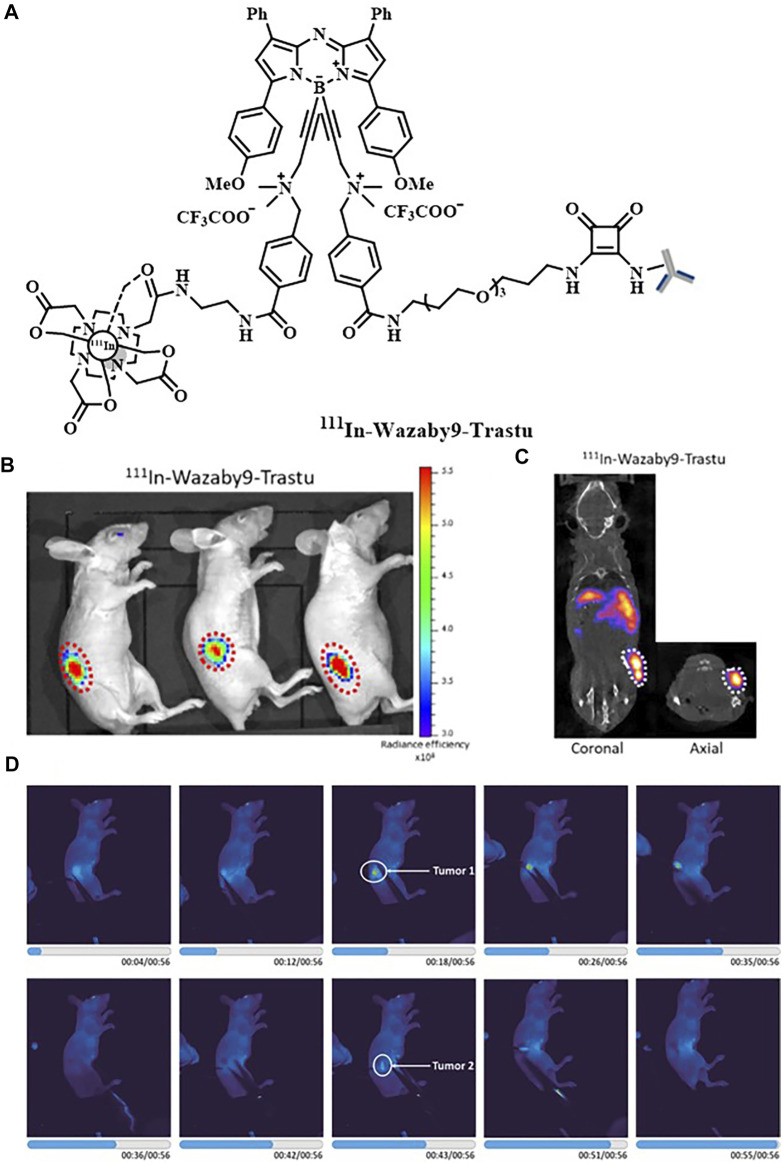
**(A)** Structure of ^111^In-Wazaby9-Trastu. **(B)** Near-infrared fluorescence images of ^111^In-Wazaby9-Trastu on mice bearing subcutaneous tumor of HCC1954 cells HER-2^+^. **(C)** Representative SPECT/CT images. **(D)** Fluorescence-guided resection of tumor tissues. Reproduced with permission from Privat et al. (2021). Copyright 2021, American Chemical Society.

Owing to the high affinity and specificity of antibodies, the imaging of labeled antibodies or engineered antibody fragments, which refers to immuno-imaging, has been widely applied in FI/SPECT bimodal probes. Hekman et al. had presented an FI/SPECT imaging probe, ^111^In-DTPA-labetuzumab-IRDye800CW, to detect carcinoembryonic antigen (CEA)–expressing pulmonary micrometastases. The near-infrared fluorescence (NIRF) dye IRDye800CW was selected, and the humanized anti-CEA monoclonal antibody, labetuzumab, was used to ensure adequate targeting of a tumor-associated antigen. Based on their experimental data, a feasibility study of this probe in patients with peritoneal carcinomatosis of colorectal origin could be considered (Hekman et al., 2017b). Besides, they replaced labetuzumab with farletuzumab and developed a new probe, ^111^In-DTPA-farletuzumab-IRDye800CW, for the intraoperative detection of ovarian cancer lesions (Hekman et al., 2017a). Later, they had also utilized immuno-imaging to design another probe ^111^In-DOTA-girentuximab-IRDye800CW. Successful visualization of tumors during surgery was performed in patients with clear cell renal cell carcinoma (ccRCC). Most notably, it was the first clinical application of tumor-targeted dual-modality imaging guided by monoclonal antibody (Figure 1) (Hekman et al., 2018). Meanwhile, boron-dipyrromethene (BODIPY) with high photoluminescence quantum yields and stability in the physiological environment was utilized to obtain ^111^In-Wazaby9-Trastu. As BODIPY was usually hydrophobic and water-insoluble, Privat et al. had succeeded in improving its solubility in aqueous media by substituting fluorine atoms on the boron by ammonium groups. Trastuzumab was bioconjugated to the probe for its high affinity toward the HER-2 receptors. ^111^In-Wazaby9-Trastu had been proved as a new tool for FI/SPECT imaging-guided surgery in nude mice bearing HER2-overexpressing HCC1954 human breast cancer xenografts (Figure 2) (Privat et al., 2021). Furthermore, the non-covalent interaction between biotin and avidin in the biotin–avidin system (BAS) is the strongest *in vivo*, which is much higher than the affinity between the antigen and antibody. Based on this, Dong et al. designed a novel dual-modality probe ^99m^Tc-HYNIC-lys(Cy5.5)-PEG_4_-biotin by using Cy5.5 as an NIR fluorophore to achieve modest signal amplification, further promoting the imaging of human colon adenocarcinoma xenografts. The *in vitro* and *in vivo* results reflected that FI exhibited exquisite congruence to SPECT imaging (Dong et al., 2016). Additionally, α_v_β_3_-integrin is an ideal tumor target spot as well, attributed to the selective attachment of RGD-containing peptides toward it. In this context, Yin et al. reported a ^125^I-radiolabeled probe cRGD-QC by which metastatic lymph nodes could be detected specifically and accurately. The probe was constructed *via* the attachment of an NIR dye (Cy5) and a quencher (QSY21) to a ^125^I-labeled cRGD. Only upon the proteolytic cleavage by activated matrix metalloproteinase-2 (MMP-2), the fluorescence of cRGD-QC was recovered. Under this circumstance, cRGD-QC showed high tumor-to-background ratios with low impact on normal tissues (Yin et al., 2019). Rizvi et al. had developed a Cy5.5-conjugated self-assembled peptide nanoprobe Cy5.5@SAPD-^99m^Tc based on a novel head-to-tail cyclic RGD-KLAK heptapeptide sequence. The effectiveness and efficacy of this probe for the diagnosis of glioblastoma multiforme had been proven in the tumor-bearing female Balb/c mice models guided by FI/SPECT imaging (Rizvi et al., 2021).

Other nanoparticles applied in FI/SPECT imaging were mainly designed by using different nanoplatforms. By combining a fluorescent dye (Dylight 755) with ^99m^Tc in multiple-armed DNA tetrahedral nanostructures (TDNs) to synthesize nanoprobe FA-Dy-^99m^Tc-TDN, the group of Jiang successfully realized noninvasive FI/SPECT imaging in tumor-bearing mice. In comparison with those of double-stranded DNA, the TDNs showed remarkably enhanced stability *in vitro* (Jiang et al., 2016). Furthermore, based on tumor cell–derived exosomes (TEx), ^99m^Tc-TEx-Cy7 was developed through the combination of Cy7 and ^99m^Tc. It was the first exosome-based nanoprobe for multimodal SPECT and NIRF imaging (Jing et al., 2021a). Remarkably, ^99m^Tc-based FI/SPECT imaging had already been applied in the clinic. Manca et al. had proved the excellent performance of ICG-^99m^Tc nanotop in both preoperative lymphatic mapping and intraoperative sentinel lymph node detection (Manca et al., 2021). Of note, Shih et al. prepared cetuximab/IR-780/micelles as immuno-imaging nanoparticles which further guaranteed the targeting properties for EGFR overexpression in colorectal cancers. A lipophilic dye, IR-780 iodide, was applied here to offer fluorescence imaging as well as photothermal therapy (PTT). In particular, it was a dual-isotope dynamic SPECT imaging system with the loaded dye and the micelles, respectively, radiolabeled with ^131^I and ^111^In (Shih et al., 2017).

#### 2.1.2 Fluorescence Imaging/Positron Emission Tomography Imaging

Compared to SPECT cameras, the scanners and radiotracers of PET can provide higher sensitivity. Unlike SPECT, positrons are emitted during the decay of the radionuclides, and then energy is released in the form of two 511-keV photons. These annihilation photons can be detected by PET cameras and therefore locate the approximate position of the PET radionuclide. However, only ^18^F, ^124^I, ^64^Cu, ^68^Ga, and ^89^Zr have been applied in FI/PET imaging (An et al., 2017; Ghosh et al., 2017; Wang Y. et al., 2020).

**FIGURE 3:**
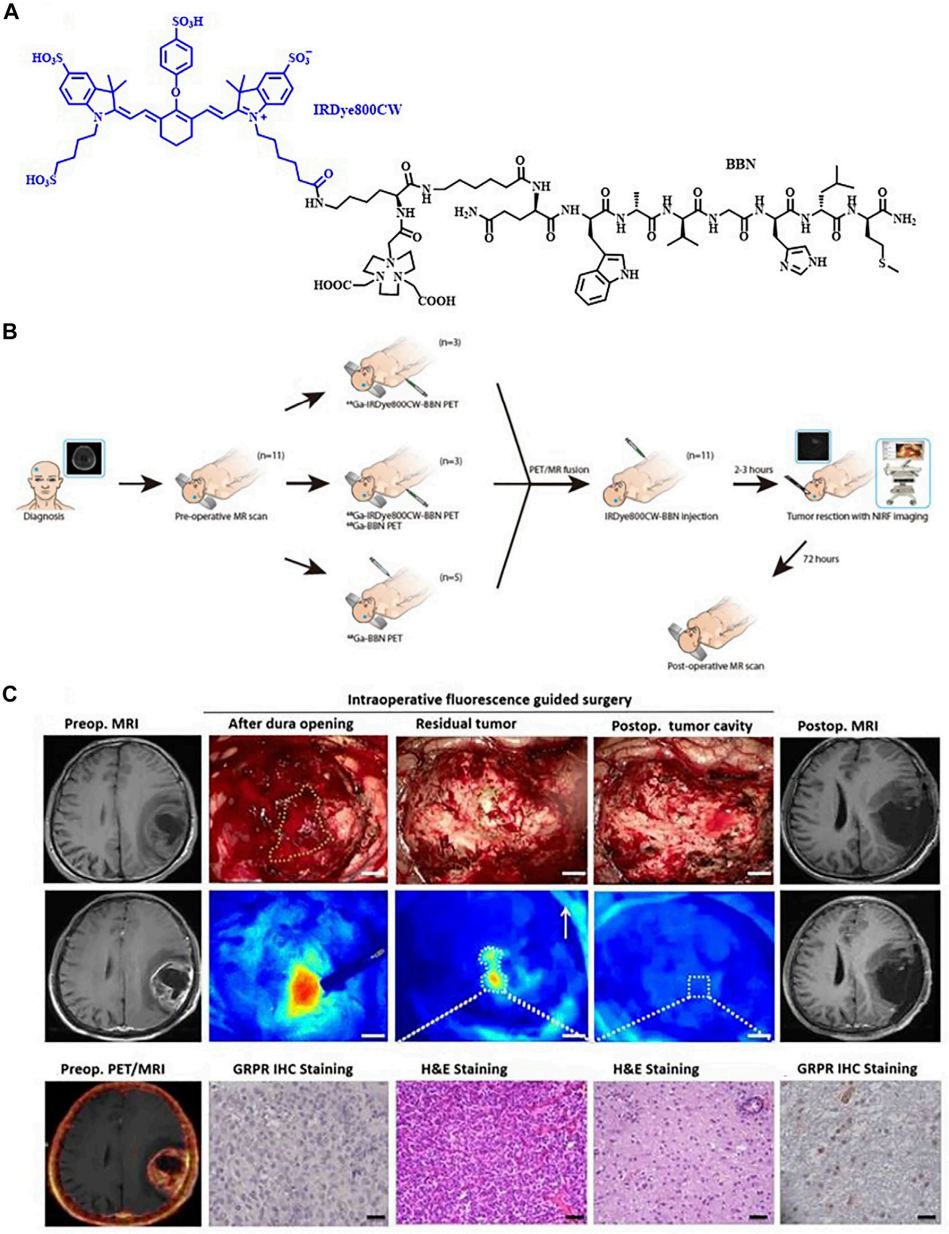
**(A)** Chemical structure of IRDye800CW-BBN tracer. **(B)** Scheme of ^68^Ga-IRDye800-BBN for the GBM patients. **(C)** ^68^Ga-IRDye800-BBN FI/PET dual-modality imaging guided GBM resection for patient 3. Reproduced with permission from Li et al. (2018). Copyright 2018, Ivyspring International Publisher.

Immuno-imaging probes at the molecular level have also been widely developed in this field. Over a third of the synthesized FI/PET bimodal probes involved antibodies (Luo H. et al., 2017). Most of these probes were labeled with radionuclides of long decay half-lives, including ^89^Zr (t_1/2_ = 3.3 d) and ^124^I (t_1/2_ = 4.2 d) which matched the pharmacokinetics of intact antibodies (Hernandez et al., 2016; Tsai et al., 2018; Zettlitz et al., 2018). Nevertheless, the use of long-lived radionuclides might result in great radiation exposure to the patient and also requires plenty of lag time before imaging. Hence, researchers focused on the design of ^64^Cu-radiolabeled probes due to the relatively long half-life of ^64^Cu (t_1/2_ = 12.7 h) among the short-lived radionuclides. For example, Adumeau et al. had presented a pre-targeted strategy for the bimodal FI/PET imaging of colorectal carcinoma. The NIR fluorophore-labeled antibody huA33-Dye800-TCO and the radioisotope ^64^Cu-Tz-SarAr were injected separately. Then, a click ligation of these two components occurred *in vivo* based on an extraordinarily rapid and biorthogonal inverse electron demand Diels–Alder reaction. As a result, little fluorescence in healthy organs and high tumor-to-normal tissues radiant efficiency ratio showed up (Adumeau et al., 2016). However, ^68^Ga had not been applied in monoclonal antibody-based FI/PET imaging owing to its extremely short half-life (^68^Ga, t_1/2_ = 67.7 min). Still, it had been widely used in the dual-modal probes for FI/PET imaging due to its relatively low cost and easy preparation from a ^68^Ge/^68^Ga generator system (Summer et al., 2017; Zhang et al., 2018). For instance, the group of Li reported a novel probe ^68^Ga-IRDye800CW-BBN. It was the first-in-human study of dual-modal FI/PET imaging to guide surgery in glioblastoma. Among the 14 patients enrolled, preoperative positive PET uptake had an excellent correlation with intraoperative NIRF signal. Patient 3 was taken as an example where even in the deep tumor cavity, the residual tumor had obviously differentiated from the adjacent normal brain tissue by dual-modality imaging (Figure 3) (Li et al., 2018).

**FIGURE 4:**
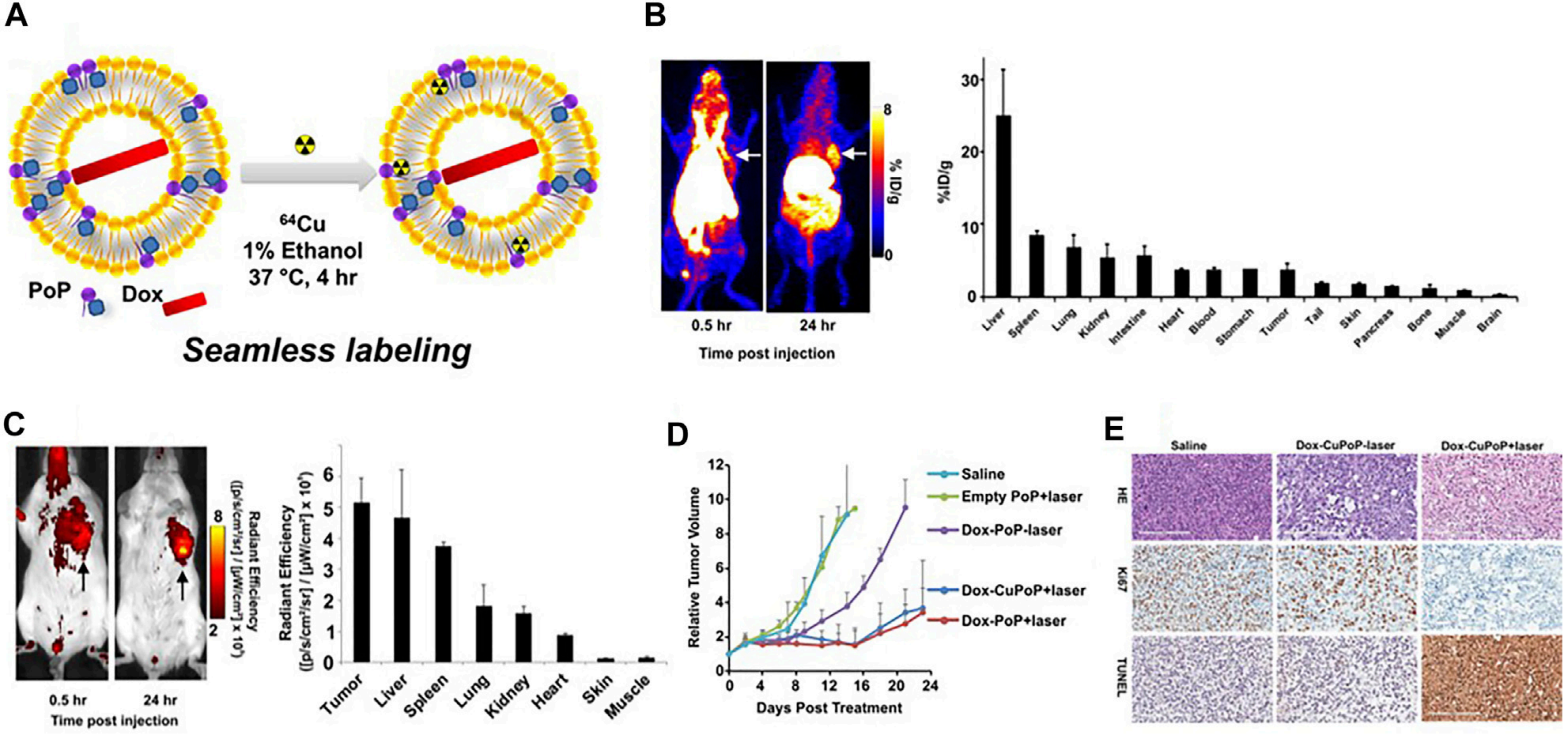
**(A)** Preparation of Dox-loaded CuPoP liposomes. **(B)** *In vivo* PET imaging and gamma counting–based biodistribution of Dox-CuPoP. **(C)** FI and quantitative fluorescence signal of mice bearing orthotopic 4T1 mammary tumors using Dox-CuPoP. **(D)** Relative tumor growth in mice bearing orthotopic 4T1 tumors after different treatments. **(E)** HE, Ki67, and TUNEL staining of tumor slices from mice receiving the indicated treatments. Reproduced with permission from Luo D. et al. (2017). Copyright 2017, American Chemical Society.

Nanoprobes were used more frequently in FI/PET imaging and mostly radiolabeled with ^64^Cu (Wang X. et al., 2018). Luo et al. developed a doxorubicin-loaded porphyrin–phospholipid (PoP) liposomes platform with intrinsic fluorescence capacity. And then ^64^Cu was radiolabeled to the stable bilayer of preformed Dox-loaded PoP liposomes to synthesize Dox-CuPoP. According to PET and fluorescent imaging, passive nanoprobe accumulation was visualized in orthotopic mammary tumor-bearing mice. In addition, the growth of 4T1 orthotopic tumors was strongly inhibited in a single chemophototherapy treatment with Dox-CuPoP under the illumination of 665 nm light (200 J/cm^2^) (Figure 4) (Luo D. et al., 2017). Du et al. had designed a breast tumor–targeted nanoprobe, PD-1-Liposome-DOX-^64^Cu/IRDye800CW, based on liposomes as well (Du et al., 2017). Besides, gold nanoparticles and polysaccharide dextran were also served as nanocarriers for ^64^Cu in FI/PET imaging (Pretze et al., 2019; Deng et al., 2020). Significantly, to further reduce potential radiation damage, Jing et al. selected extracellular vesicles derived from adipose-derived stem cells (ADSCs) as nanoplatforms, and Cy7 was afforded to obtain Cy7-EV-N_3_. ^68^Ga was connected to the platform based on the click reaction between Cy7-EV-N_3_ and ^68^Ga-L-NETA-DBCO *in vivo*. Comparing different pre-targeting and imaging time points, maximum tumor uptake was obtained at 20 h after the injection of Cy7-EV-N_3_ and at 2 h after the injection of ^68^Ga-L-NETA-DBCO. The radiation exposure was obviously reduced based on this strategy (Jing et al., 2021b).

**FIGURE 5:**
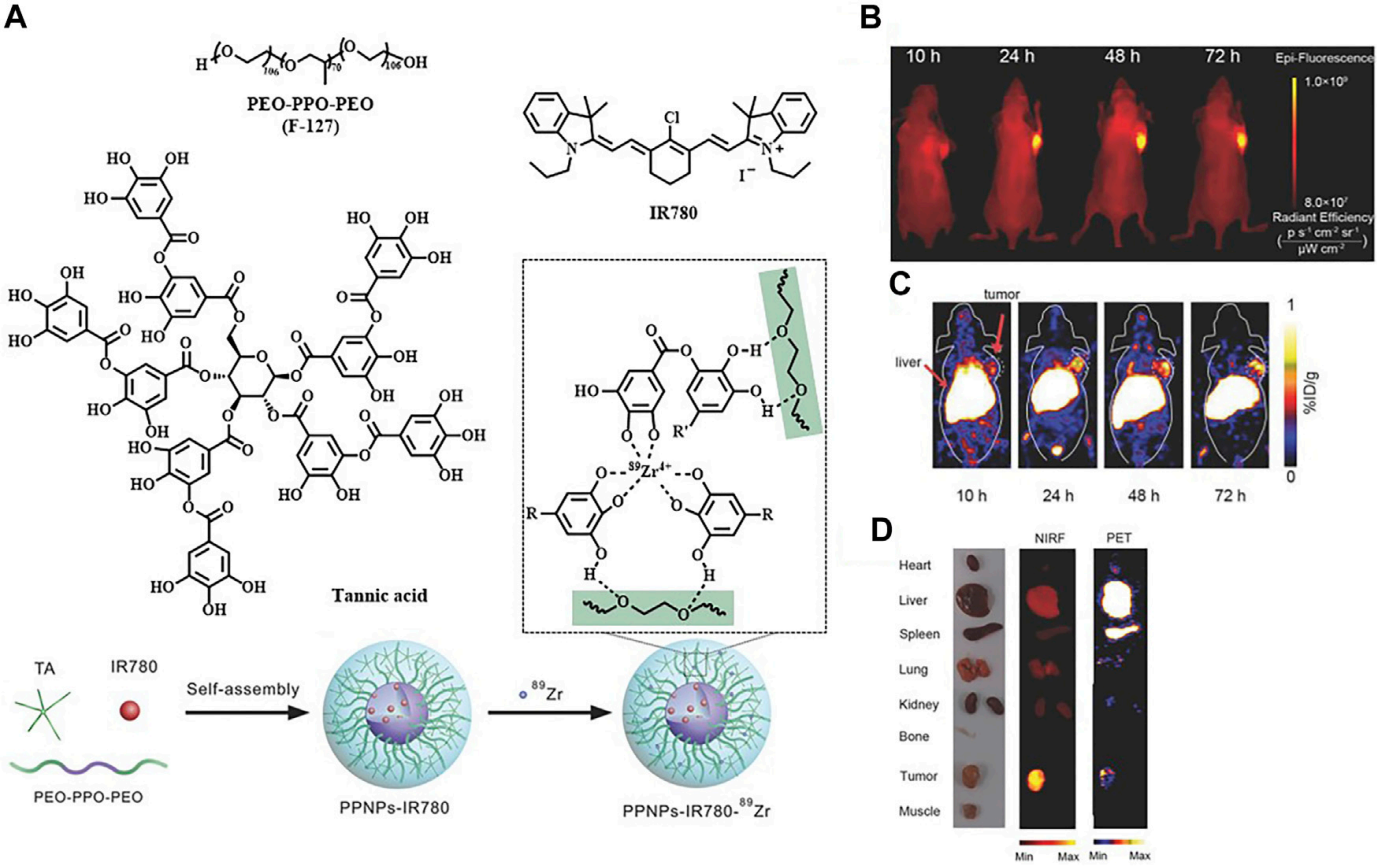
**(A)** Construction of PPNPs-IR780-^89^Zr. **(B)** NIR fluorescence images of a SKBR3 tumor bearing mouse by post–intravenous injection of PPNPs-IR780. **(C)** Serial coronal PET images. **(D)** *Ex vivo* biodistribution. Reproduced with permission from Wang X. et al. (2018). Copyright 2018, Wiley-VCH.

**FIGURE 6:**
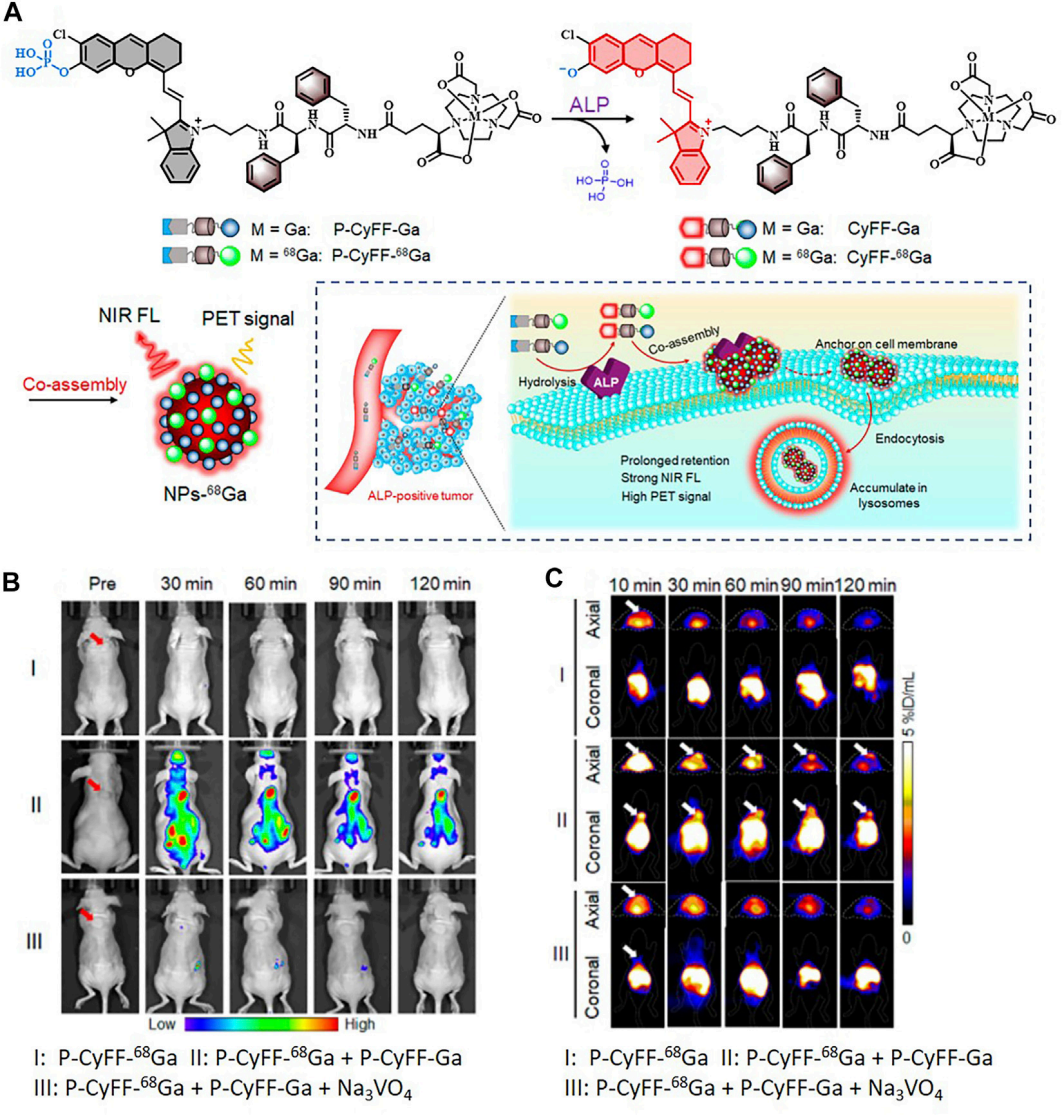
**(A)** Schematic illustration showing an ALP-enabled *in situ* co-assembly strategy for FI/PET bimodal imaging. **(B)** Fluorescence images of HeLa tumor–bearing mice with different treatments. **(C)** PET imaging of HeLa tumor in mice at 10, 30, 60, 90, and 120 min postinjection of indicated substance. Reproduced with permission from Hu et al. (2021). Copyright 2021, American Chemical Society.

Meanwhile, there remain challenges that nanoprobes often suffer from poor reproducibility and low tumor penetrability due to their much larger sizes compared to organic small-molecule probes (Yan et al., 2019). The group of Wang considered designing self-assembled nanoparticles to weaken the effect of these defects. The polyphenol and poloxamer self-assembled supramolecular nanoparticles (PPNPs) were fabricated by multivalent hydrogen bonding between tannic acid and Pluronic F-127 together with hydrophobic interactions of poly(propylene oxide) chains. IR-780 was encapsulated by PPNPs through hydrophobic interactions to perform NIRF imaging after releasement. Then PPNPs-IR-780 was labeled with ^89^Zr to form PPNPs-IR-780-^89^Zr without additional chelators, which was attributed to the excess phenolic hydroxyl groups of tannic acid. Of note, the *in vivo* NIR fluorescent images showed surprisingly higher fluorescence intensity in tumors than in other tissues, while the vast majority of PPNPs-IR-780-^89^Zr was accumulated in the liver and spleen according to PET imaging. As the leakage of IR-780 from the probe could be observed in serum, it might explain why FI and PET imaging showed different biodistribution of the probe (Figure 5) (Wang X. et al., 2018). Additionally, Hu et al. reported an enzyme-activatable probe P-CyFF-^68^Ga and its cold probe (P-CyFF-Ga) which resulted in co-assembling into fluorescent and radioactive nanoparticles (NP-^68^Ga) in response to alkaline phosphatase (ALP). It was demonstrated that the ALP-triggered *in situ* formed NP-^68^Ga was capable of providing FI/MRI of the ALP-positive tumors with high sensitivity and deep penetration depth (Figure 6) (Hu et al., 2021).

### 2.2 Fluorescence Imaging/Magnetic Resonance Imaging

By recording the spatial distribution of water protons, MRI is capable of generating a 3D anatomical image of tissues. It has been broadly applied in the aspect of soft tissue lesions and deep tissue pathological details imaging (Domey et al., 2016; Tsai et al., 2018). Further compared with NMI, the images can be formed without injecting radionuclide which provides higher safety (Kim et al., 2016). The signal intensity of MRI is affected by the relaxation rate of water protons which includes the longitudinal relaxation rate and transverse relaxation rate. Contrast agents (Cas) used in MRI are to shorten the longitudinal relaxation time (*T*_1_) or the transverse relaxation time (*T*_2_) in order to image specific regions of interest. Hence, they can be divided into *T*_1_-weighted Cas [e.g., paramagnetic gadolinium (Gd) or manganese (Mn)] and *T*_2_-weighted Cas [e.g., superparamagnetic iron oxide (SPIO)]. *T*_1_-weighted Cas shows positive contrast enhancement with brighter images by shortening the *T*_1_ of protons, and *T*_2_-weighted Cas shows negative contrast enhancement with darker images by shortening the *T*_2_ of protons. However, MRI has some constraints such as poor resolution and lack of sensitivity because of the overlap of *T*_1_ or *T*_2_ between healthy tissues and lesions. As an ideal complement to MRI, FI can provide sensitive imaging to distinguish pathological organs with high temporal resolution. The development of FI/MRI probes allows the imaging of the fine distribution of the probes in tissues and provides various information, including anatomical, physiological, and even molecular information (Ortgies et al., 2016; Getachew et al., 2022).

Beyond the probe Gd-Cy7-PTP/RGD designed by the group of Wang for pancreatic ductal adenocarcinoma (PDAC) imaging (Wang Q. et al., 2018), other FI/MRI probes were platform-free or platform-based nanoprobes (Supplementary Table S1). Platforms, such as mesoporous silica, serum albumin, micelles, and liposomes, had been widely applied in this field which were mainly involved in *T*_1_-weighted Cas-contained probes (Cressey et al., 2021).

**FIGURE 7:**
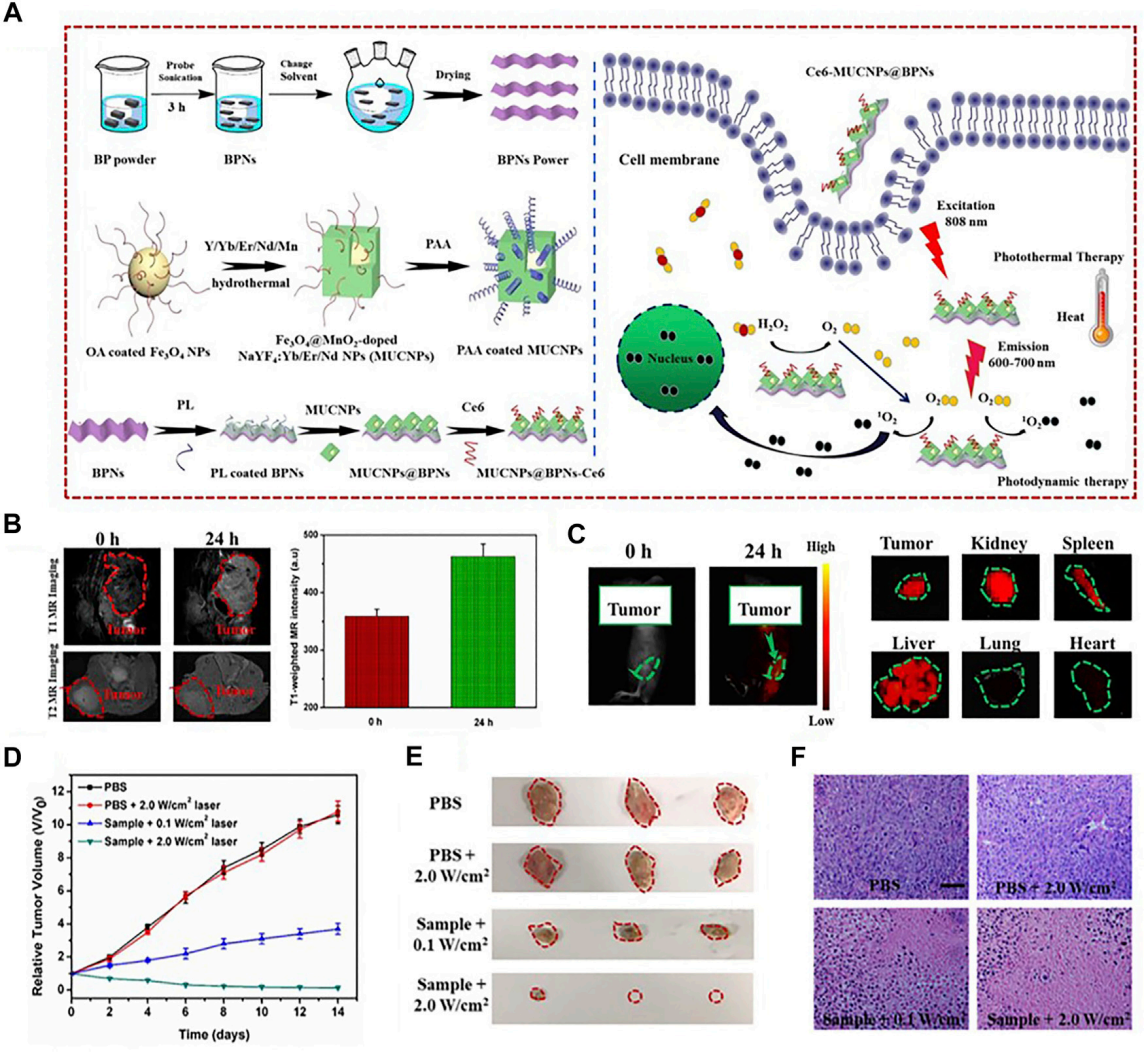
**(A)** Schematic illustration of the fabrication of MUCNPs@BPNs-Ce6 and its antitumor therapy. **(B)** *In vivo* MR images and *T*_1_-weighted MR signals in the tumor before and after injection with MUCNPs@BPNs-Ce6 for 24 h. **(C)** *In vivo* and representative *ex vivo* fluorescence images of Hela tumor–bearing mice. **(D)** *In vivo* tumor volume curves of tumor-bearing mice in different groups after various treatments indicated. **(E)** Photographs of excised tumors. **(F)** HE staining of tumor tissue (scale bar = 100 μm). Reproduced with permission from Zhang et al. (2020). Copyright 2020, Elsevier.

One widely used strategy to develop dual-modal imaging agents for FI/MRI is based on fluorescent labeling magnetic nanoparticles (MNPs). MNPs not only possess controllable dimensions and easily modified surfaces but can also provide excellent magnetic responsiveness which is manipulated by external magnetic fields. In particular, iron oxide nanoparticles have drawn much attention due to their low toxicity and feasibility to produce iron oxide cores (Lee et al., 2018; Li et al., 2019a; Reichel et al., 2020). Many hydrophilic substances [such as polyethylene glycol (PEG), poly(acrylic acid) (PAA), galactosyl conjugated P_123_ (Gal-P_123_), and red blood cell membrane] were used to modify iron oxide NPs in order to enhance biocompatibility and stability (Liang et al., 2017; Wang et al., 2020a). Polymers, such as polyamidoamine (PAMAM) and poly(lactic-co-glycolic) acid (PLGA), were ideal candidates to label NIR fluorophores (Duan et al., 2019; Wang et al., 2019d). Moreover, there were some other substances, such as calcium carbonate, dextran, chitosan, poly(succinimide) (PSI), and liposome, that could combine the ability of improving biocompatibility and encapsulating fluorescent molecules (Yang et al., 2018; Xie et al., 2020). For example, Zhang et al. had designed a novel theranostic nanocomposite MUCNPs@BPNs-Ce6. To prepare MUCNPs, the red upconversion luminescent shell (MnO_2_-doped NaYF_4_:Yb/Er/Nd) was performed on the oleic acid capped Fe_3_O_4_ NPs by hydrothermal synthesis. The upconversion part was used to match the wavelengths of black phosphorus (BP) for making optimal PTT and photodynamic therapy (PDT) work simultaneously. Afterward, MUCNPs were modified with PAA to acquire good hydrophilicity. MUCNPs@BPNs-Ce6 was finally obtained through the coupling between the carboxyl groups of the chlorin e6 (Ce6) molecules and the exposed amino groups of MUCNPs. The results demonstrated that the probe could be exploited as a good theranostic agent for simultaneous FI/MRI, highly efficient PDT and PTT of tumor (Figure 7) (Zhang et al., 2020). Besides, dozens of SPIOs were encapsulated in one nanocapsule (NC) *via* an adapted water-in-oil-in-water (W/O/W) emulsion by poly(ε-caprolactone-co-lactide)-β-poly(ethylene glycol)-b-poly(ε-caprolactone-co-lactide) (PCLA-PEG-PCLA) polymers through a solvent evaporation process, reported by Liao et al. A cyanine dye IR-820 and paclitaxel (PTX) were introduced into the NCs to develop NC-SPIOs-IR-820-PTX. It could provide FI/MRI, as well as chemotherapy and PDT, for 4T1 tumors of mice (Liao et al., 2017). The group of Wang also applied the W/O/W emulsion method to co-load doxorubicin (DOX) and indocyanine green (ICG) into Fe/FeO-PPP heterostructures to form DOX-ICG@Fe/FeO-PPP nanocapsules. The designed nanoprobe was capable of dual-imaging of the KB tumor-bearing nude mice (Wang Z. et al., 2019).

The method of constructing fluorescent-labelling MNPs in *T*_2_-weighted bimodal FI/MRI has also been used as a reference in the design of *T*_1_-weighted dual-modal nanoprobes. However, for the synthesis of these probes, the formation of *T*_1_-weighted nanoprobes based on different nanoplatforms was required at first. Sahu et al. selected serum albumin as a surface stabilizer to produce Prussian blue (PB) nanoparticles. And then, ICG was encapsulated into the PB-BSA nanoparticles to form PB-BSA-ICG. Even if PB had the ability to shorten *T*_1_ and *T*_2_, they only focused on the *T*_1_-weighted MRI property of the probe. PB-BSA-ICG could provide bimodal FI/MRI of the tumor as well as a combined PTT-PDT (Sahu et al., 2016). The other *T*_1_-weighted dual-modal nanoprobes were based on Gd or Mn which depended on platforms including serum albumin, regenerated silk fibroin, micelle, monolayered-double-hydroxide nanosheets, carbon cage, and mesoporous silica (Picchio et al., 2021). Among them, taking the most commonly used platform, serum albumin, as an example, the group of Hao integrated ICG and PTX into the albumin corona of Gd_2_O_3_@HSA nanoparticles to prepare PIGH NPs. The experimental results of the fluorescence and MR bimodal imaging showed a high 4T1 tumor accumulation. The potential of chemotherapy and PTT of PIGH NPs was proven as well (Hao et al., 2019). Besides, it is worth noting that some nanoprobes were synthesized by utilizing the opposite form of this method. They were constructed through CAs-labeling fluorescent NPs (Park et al., 2017; Ma et al., 2021; Yang et al., 2022).

Another common strategy is to integrate NIR fluorophore and CA based on the nanoscale matrix, including glycan, protein or polylysine, DNA bipyramid nanostructure, virus-like particles, liposomes, silica-based NPs, gold nanocarrier, and PLGA NPs (Key et al., 2016; Henderson et al., 2018; Sasikala et al., 2018). Nonetheless, the effect of aggregation fluorescence quenching (ACQ) in a majority of traditional organic fluorophores has extensively limited their biomedical applications. ACQ represented that in high concentration solutions or in the aggregate state, some fluorophores possessed faint or annihilated emission. Aggregation-induced emission (AIE), defined by the group of Luo in 2001, provided a straightforward solution for the problem of ACQ (Luo et al., 2001; Xu et al., 2021). In this scenario, triphenylamine-divinylanthracene-dicyano (TAC), an aggregation-induced emission fluorogen (AIEgen), was introduced by the group of Ma to develop a PLGA NPs–based nanoprobe anti-VEGF/OA-Fe_3_O_4_/TAC@PLGA. TAC and Fe_3_O_4_ were loaded into the PLGA NPs, while anti-VEGF antibody was afforded to functionalize the surface of PLGA NPs for specifically targeting cancers with overexpressed VEGF-A. It was demonstrated that this system showed desirable NIR-emission as well as suitable magnetic properties for ultrasensitive localization of cancer cells (Ma et al., 2019).

**FIGURE 8:**
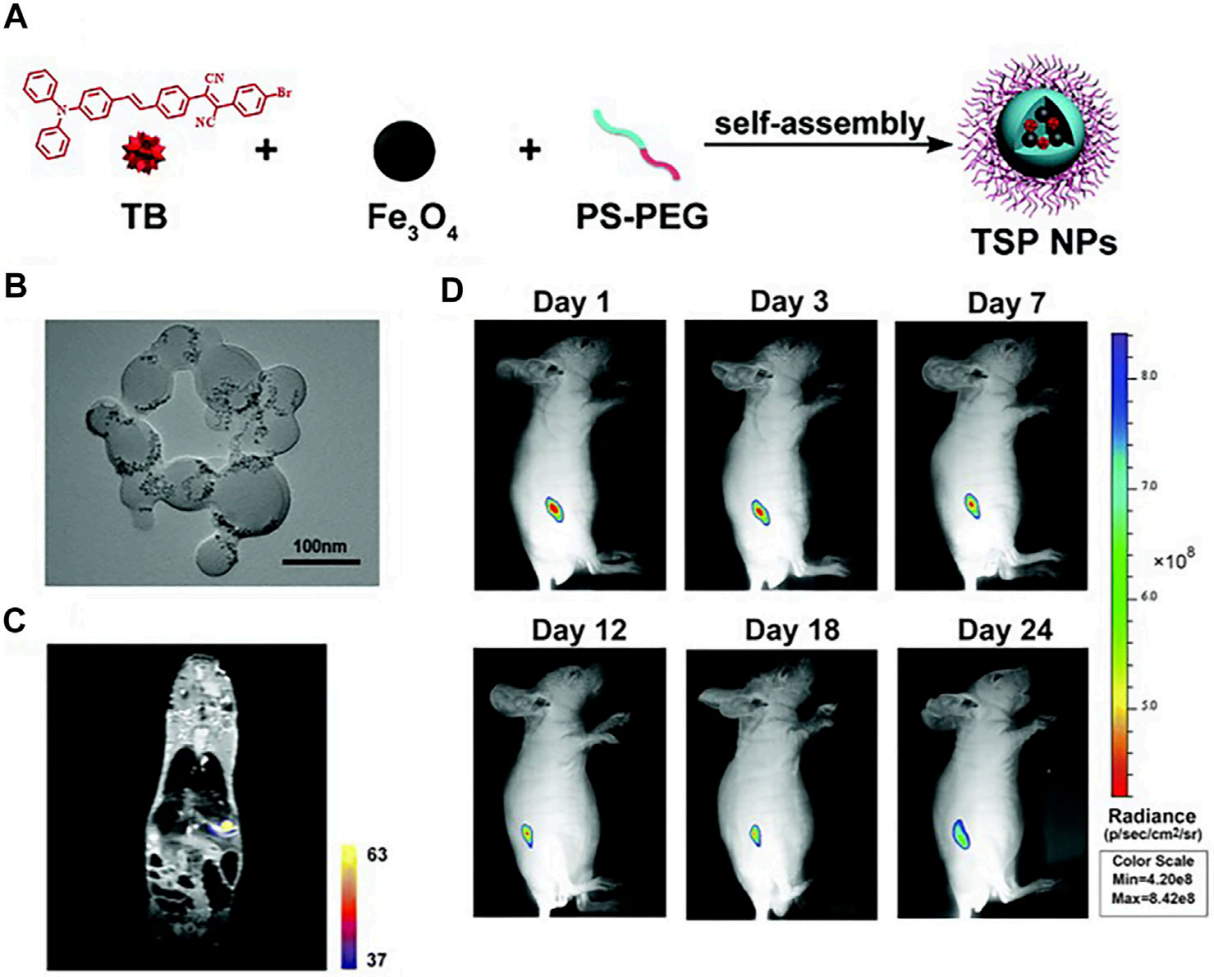
**(A)** The fabrication of TSP NPs. **(B)** TEM image of TSP NPs. **(C)** MPI-MRI fusion images *in vivo*. **(D)** Representative *in vivo* fluorescence images of the mouse subcutaneously injected with HuH-7 cells from day 0 to day 24. Reproduced with permission from Meng et al. (2019). Copyright 2019, Royal Society of Chemistry.

Additionally, self-assembled nanoprobes have been widely reported in the field of dual-modal FI/MRI. Wu and the co-workers reported a self-assembled probe ICG-FA-PPD which was constructed through electrostatic interaction. The positively charged section was provided by FA-PPD which was prepared through folic acid (FA) and Gd-DOTA–modified polyethylenimine-PEG, while the negative charge was offered by ICG. The two parts were assembled in pH 7–7.4 to form ICG-FA-PPD. The synthesized nanoprobe possessed the capability for simultaneous FI/MRI and PDT toward glioblastoma (Wu et al., 2016). Except for ICG-FA-PPD, most of these self-assembled probes depended on substances with a hydrophobic interior that could encapsulate NIR fluorophores and CAs to form clathrates through host–guest interactions. For example, the group of Gao developed a new type of theranostic polymer NPs which was fabricated *via* the co-assembly of amphiphilic paramagnetic block copolymers (PCL-b-PIEtMn) and polycaprolactone-b-poly(ethylene glycol) (PCL-b-PEG), in which IR-780 and DOX were co-encapsulated. The results of *in vitro* and *in vivo* experiments proved the ability of the probe in simultaneous FI/MRI, and the synergistic effect of PTT with chemotherapy (Gao et al., 2020). Meanwhile, AIE had been reported in this field as well. Meng et al. synthesized a novel bimodal FI/MRI nanoprobe TB/SPIO@PS-PEG nanoparticles (TSP NPs). The AIEgen, 2-(4-bromophenyl)-3-(4-(4-(diphenylamino)styryl)phenyl)fumaronitrile (TB), and SPIO were cooperatively self-assembled with polystyrene-polyethylene glycol (PS-PEG) to obtain TSP NPs. Multimodal imaging revealed that TSP NPs could provide deep penetration images and detailed anatomical features of liver tumor *in situ* (Figure 8) (Meng et al., 2019).

**FIGURE 9:**
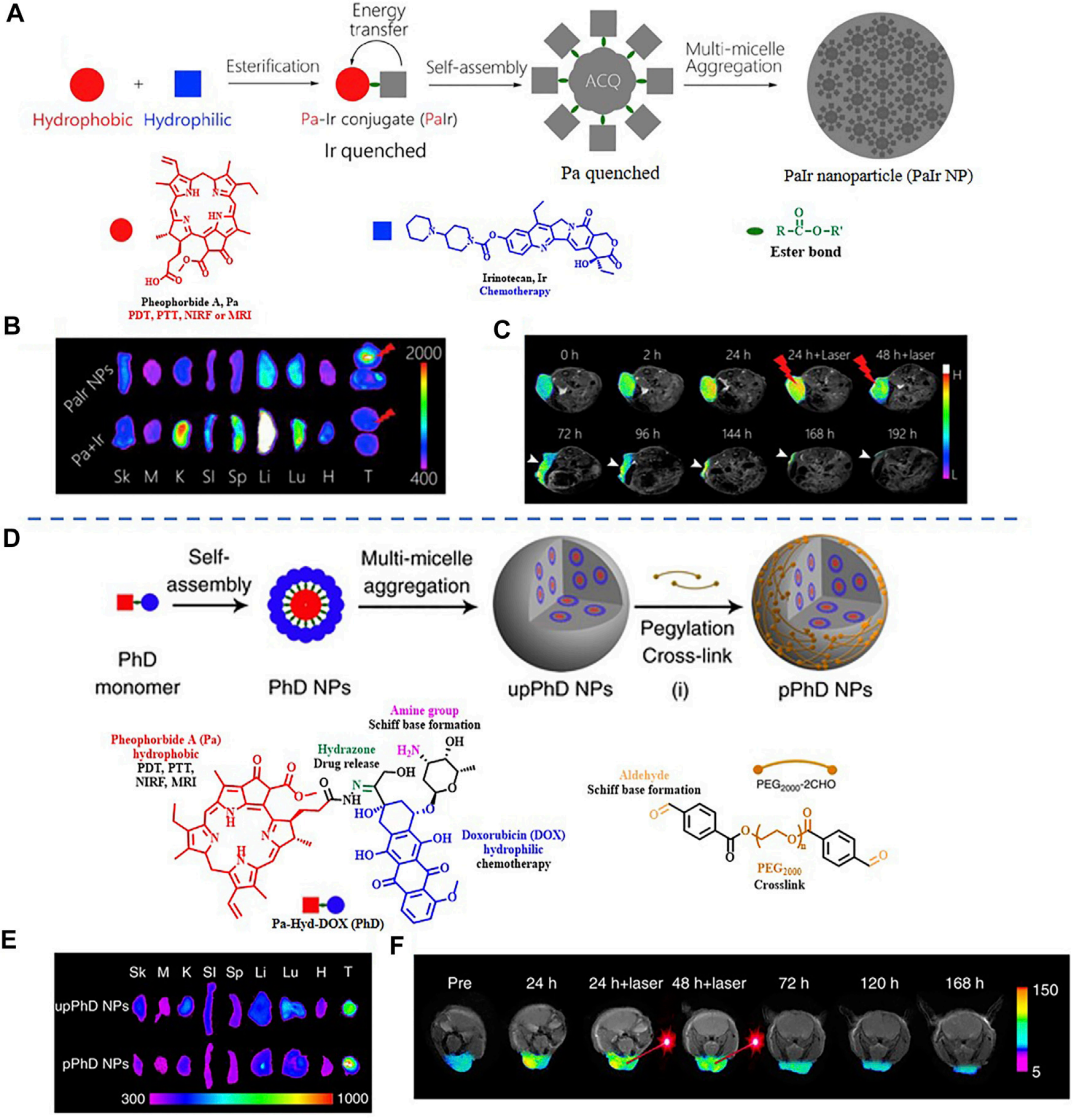
**(A)** The fabrication of PaIr NPs. **(B)** *Ex vivo* distribution of PaIr NPs indicated by intrinsic NIRF. **(C)** Self-indication of the phototherapeutic effect by *T*_1_-weighted MRI. Reproduced with permission from Xue et al. (2018b). Copyright 2018, Elsevier. **(D)** Schematic illustration of the fabrication of pPhD NPs. **(E)** *Ex vivo* distribution of pPhD NPs indicated by intrinsic NIRF. **(F)** Phototherapeutic effect monitored by MRI. Reproduced with permission from Xue et al. (2018a). Copyright 2018, Nature.

**FIGURE 10:**
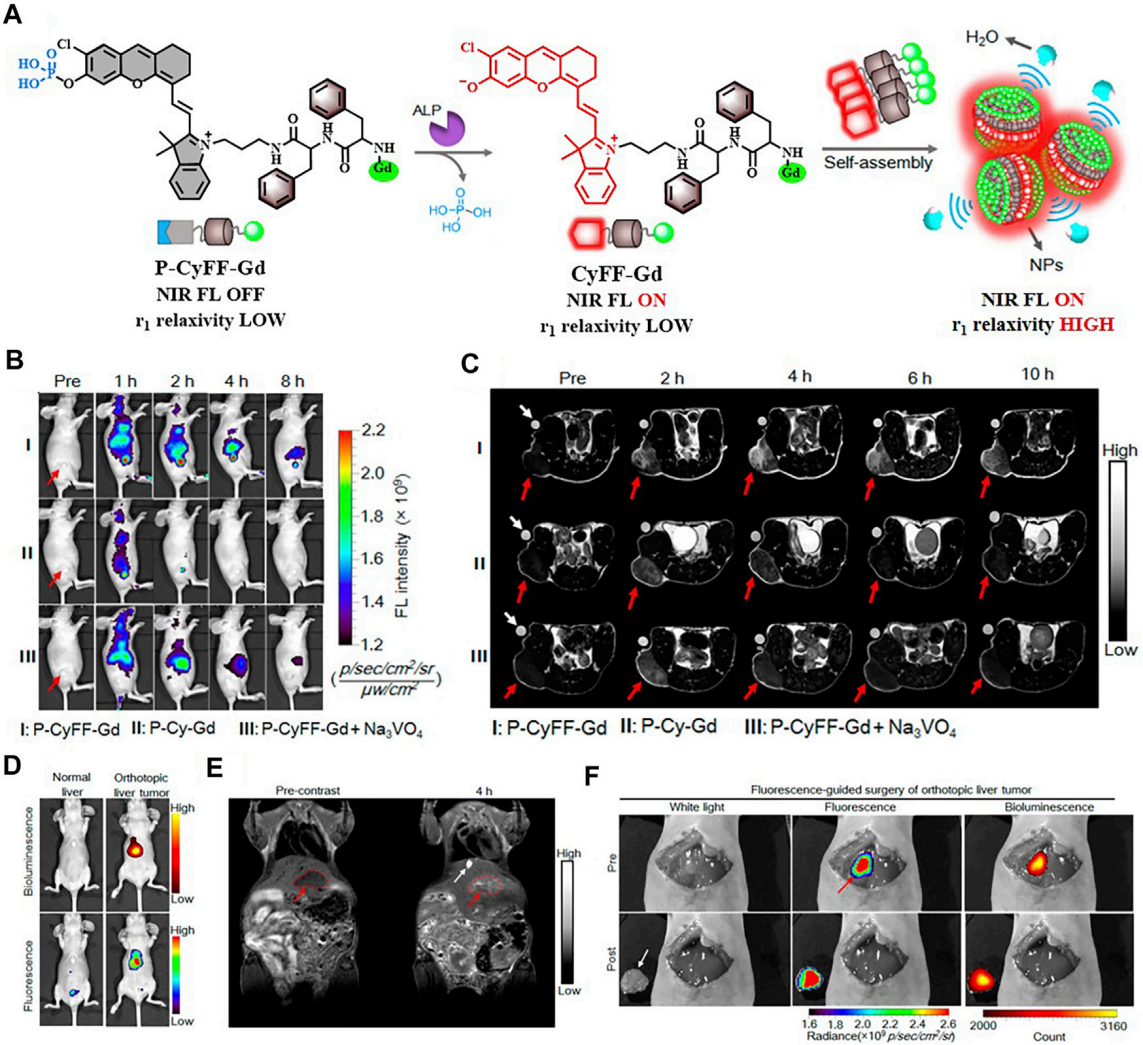
**(A)** Schematic illustration of P-CyFF-Gd *in situ* self-assembly. **(B)** Longitudinal FI of mice after various treatments is indicated. **(C)** *T*_1_-weighted MRI of HeLa tumor-bearing mice receiving different treatments. **(D)** Whole-body fluorescence (down) and bioluminescence (up) imaging of normal mice and orthotopic HepG2/Luc liver tumor xenograft mice following i.v. injection of P-CyFF-Gd (50 μM, 200 μl) at 4 h **(E)** *T*_1_-weighted MRI of orthotopic HepG2/Luc liver tumor xenograft mice before and after i.p. injection of P-CyFF-Gd (0.015 mmol/kg). **(F)** Imaging-guided surgical resection of orthotopic HepG2/Luc liver tumor in an intraoperative mouse 30 min after directly spraying P-CyFF-Gd (10 μM) on the liver. Reproduced with permission from Yan et al. (2019). Copyright 2019, American Chemical Society.

It is worth noting that some dual-modal FI/MRI nanoprobes could self-assemble into NPs without using inert nanomaterials. The group of Xue had presented two kinds of fully active pharmaceutical ingredient nanoparticles (FAPIN), PaIr NPs, and PhD NPs, in succession for different tumors imaging. The two reported FAPIN were self-assembled by minimal materials, but seamlessly orchestrated versatile theranostic functionalities including self-delivery, self-fluorescence/MR indicating, and tri-modality cancer therapy (Figure 9) (Xue et al., 2018a; Xue et al., 2018b). Moreover, Yan et al. synthesized an enzyme-activatable *in situ* self-assembled FI/MRI bimodal probe P-CyFF-Gd. In response to ALP, the fluorescence signal of the probe was recovered followed by self-assembly, leading to increased relaxivity (r_1_). The experimental data indicated that P-CyFF-Gd was a noninvasive FI/MRI probe which could locate and guide the real-time surgical resection of orthotopic liver tumor (Figure 10) (Yan et al., 2019).

### 2.3 Fluorescence Imaging/Computed Tomography Imaging

CT, which can form 3D visual reconstruction of interested tissues given by the different X-rays attenuation degrees of different tissues, is a powerful noninvasive imaging technology with advantages of high spatial resolution, accurate anatomical information, and relatively affordable price (Murakami and Nakahara, 2014). To further increase the visibility of internal body structure, CAs used in CT are required to provide different X-rays attenuation degrees from the surrounding organs (Nunez et al., 2020). X-rays are thereby more or less absorbed by them than by body constituents, particularly by water. Materials containing elements with high atomic numbers and proper X-ray attenuation coefficients, such as iodine (I), bismuth (Bi), and gold (Au), have been used as CT CAs (Reimer et al., 2021; Sood et al., 2021; Tarighatnia et al., 2021). Based on the high spatial resolution of CT, FI/CT dual-modal imaging is highly complementary as FI is a real-time imaging technology with high sensitivity, and CT can provide 3D anatomic details with virtually no penetration limitations.

Some effort had been devoted to designing small-molecule probes for FI/CT imaging, instead, nanoprobes were further promoted based on different CAs. For example, the X-ray attenuation coefficient of iodine is 1.94 cm^2^/g at 100 eV, which allows for available contrast effects in CT imaging. Lee et al. designed a novel bimodal FI/CT imaging probe Cy5.5-HA-TIBA/DOX with selecting 2,3,5-triiodobenzoic acid (TIBA) as a CT imaging CA and Cy5.5 as a NIR-I fluorescence reporter. By conjugating to an HA oligomer, they could self-assemble into a nanoprobe. The abilities of tumor targeting, dual-modal imaging, and cancer diagnosis of the synthesized nanoprobe were demonstrated in an SCC7 tumor-xenografted mouse model (Lee et al., 2016). A commercially available CT CA, iohexol, also had applications in FI-based dual-modal imaging. Based on liposomes, it was co-encapsulated with ICG to form CF800 (Zheng et al., 2015). Patel et al. and Wada et al. proved the feasibility of CF800 for disease localization and FI/CT imaging in an orthotopic human NSCLC mouse model and a rabbit VX2 lung tumor model, respectively (Patel et al., 2016; Wada et al., 2019).

Compared with iodine, gold has a longer circulation time, bigger atomic number (Z = 79), and larger absorption coefficient (Au, 5.16 cm^2^g^−1^), which thus shows approximately 2.7 times higher contrast per unit mass (Xu et al., 2008). Zeng and his co-workers designed an NIR-response PTT platform (Au@MSNs-ICG) for FI/CT imaging of liver tumor. Gold nanospheres were selected to serve as CT CAs, and ICG was used to provide a fluorescent signal (Zeng et al., 2016). Furthermore, Wu et al. also reported a nanoprobe (AuNCs/PPI-ICG nanohybrid) based on the same fluorophore and CAs. It showed clear fluorescence and CT signals as well as excellent photodynamic properties and exceptional photothermal capabilities in A549 cells (Wu M. et al., 2019). Additionally, the overlap between the emission spectrum of Cy5.5 and the excitation spectra of gold NPs resulted in light quenching inside the probe GNPs-CKL-FA. Gold NPs and Cy5.5 were connected with an acid-labile ketal linker which would be hydrolyzed upon reaching acidic microenvironment. The FI/CT dual-modal imaging of this pH-activatable probe was verified in human cervical cancer xenografts (Tang et al., 2019). Besides, the absorption coefficient of bismuth (Bi, 5.74 cm^2^g^−1^) which is higher than gold such that it is even more beneficial to CT imaging. Sun and his co-workers reported a multifunctional nanocomplex (BPDC NSs) for tumor theranostics. The probe was formed through loading Ce6 and DOX in the PEGylated bismuth sulfide nanostars (Bi_2_S_3_ NSs). Ce6 served as the fluorophore and the photosensitizer, while DOX was used as a chemotherapeutic drug. It was demonstrated that BPDC NSs exhibited rapid accumulation toward tumor as evidenced by tracing the fluorescence and CT signals (Sun et al., 2019a).

More than that, many efforts have been devoted to exploring 2D transition-metal dichalcogenides (TMDs) in recent years, such as MoS_2_, WS_2_, and MoSe_2_. The majority of 2D TMDs have a strong X-ray attenuation ability. Liu et al. developed a FI/CT imaging nanoprobe (PEG-MoS_2_-Au-Ce6) for 4T1 tumors by attaching Ce6 to the gold nanoparticles (AuNPs)–decorated molybdenum disulfide (PEG-MoS_2_) nanosheets. Ce6 remained in its quenched state due to the influence of AuNPs and PEG-MoS_2_ nanosheets. And upon heat generation, it was released from the probe to regain strong fluorescence signals (Liu L. et al., 2017). While the group of Zhou attached to WS_2_ NPs to photosensitive Au_25_(Captopril)_18_-(Au_25_) nanoclusters *via* electrostatic interaction to construct WLPD-Au_25_ for the monitoring of S180 tumor-bearing mice. In WLPD-Au_25_, WS_2_ NPs were used for providing CT images and IR-783 was loaded for NIR imaging (Zhou et al., 2019). Besides, other CT CAs, including Ba^2+^, CuS NPs, and W_18_O_49_ NPs, had been applied in FI/CT imaging as well (Zeng et al., 2017; Shi et al., 2018; Yang et al., 2019).

### 2.4 Fluorescence Imaging/Photoacoustic Imaging

PAI is an emerging modality in biomedical imaging mainly based on the distinction of the photoacoustic effect between malignant and healthy tissue. When the pulsed laser is illuminated into the target tissues, the light energy they absorb can be partly transformed into heat which leads to increased temperature and further thermal elastic expansion, thereby generating the acoustic waves. However, there is little variation in the photoacoustic signals among different organs, so exogenous CAs with higher sensitivity for PAI are often required. Most of these CAs are organic and inorganic nanomaterials, such as carbon nanotubes, gold NPs, and silver NPs (De La Zerda et al., 2008; Kim et al., 2009; Homan et al., 2012). Multispectral optoacoustic tomography (MSOT) is an optoacoustic imaging technique by which the signals of CAs can be separately visualized. By employing CAs, the limitation of depth in traditional optical imaging techniques can be relatively solved. Meanwhile, PAI can also offer a high ultrasonic resolution to overcome the dilemma in resolution. Due to the advantages of PAI, the combination of FI and PAI is an effective imaging strategy with both microscopic spatial resolution and macroscopic ultrasensitivity. In addition, the majority of the dual-modal probes of FI/PAI could offer PTT toward tumors based on the mechanism of PAI.

**FIGURE 11:**
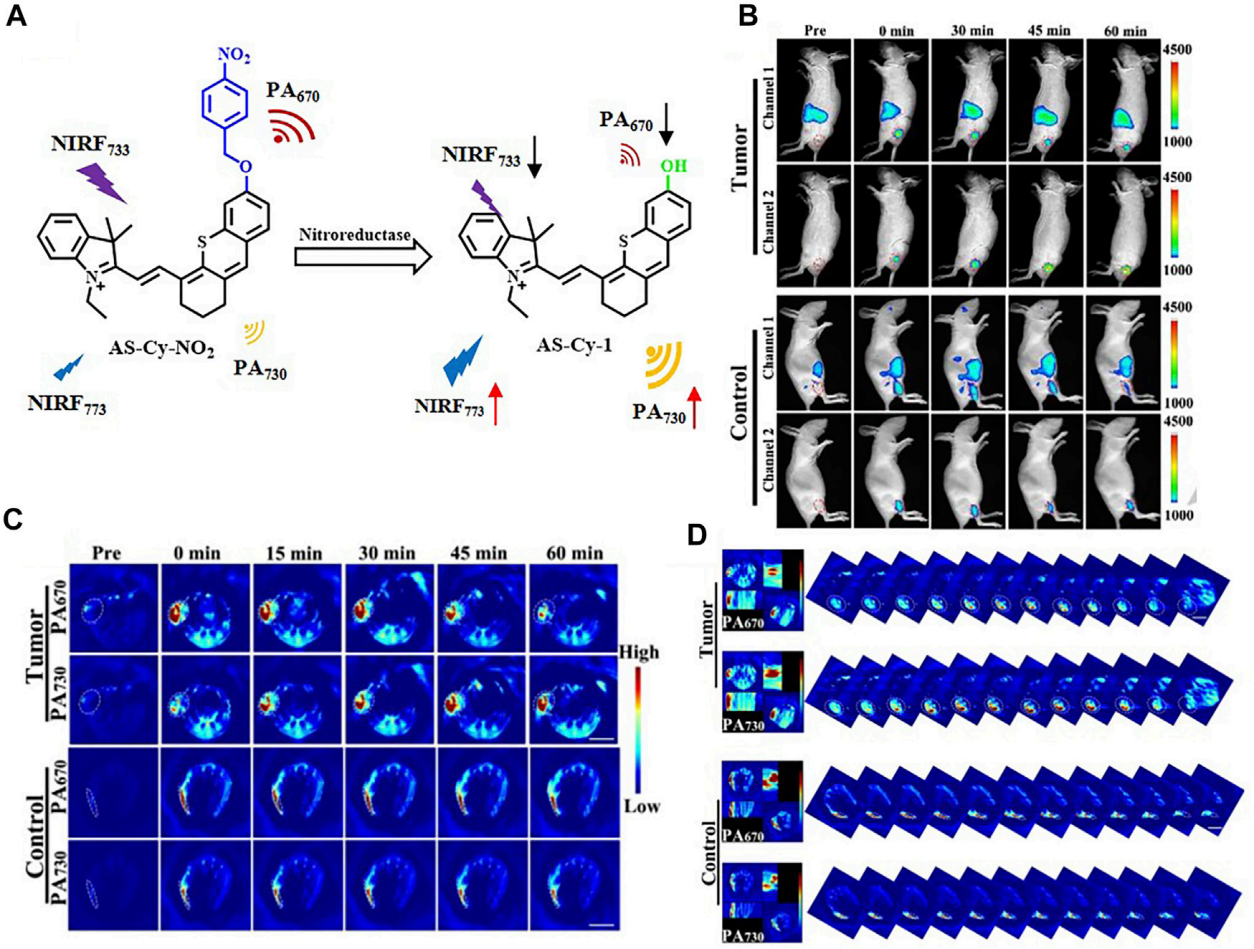
**(A)** Mechanism of AS-Cy-NO_2_ for noninvasively detecting NTR. **(B)** *In vivo* ratiometric fluorescent imaging of AS-Cy-NO_2_ for hypoxia in mice. **(C)** *In vivo* ratiometric PAI of 4T1 tumor bearing and the control hind limbs of nude mice postinjection with the AS-Cy-NO_2_. **(D)** Representative orthogonal-view 3D MSOT images covering the whole tumor region and the control hind limbs. Reproduced with permission from Zhang et al. (2022). Copyright 2022, Wiley-VCH.

**FIGURE 12:**
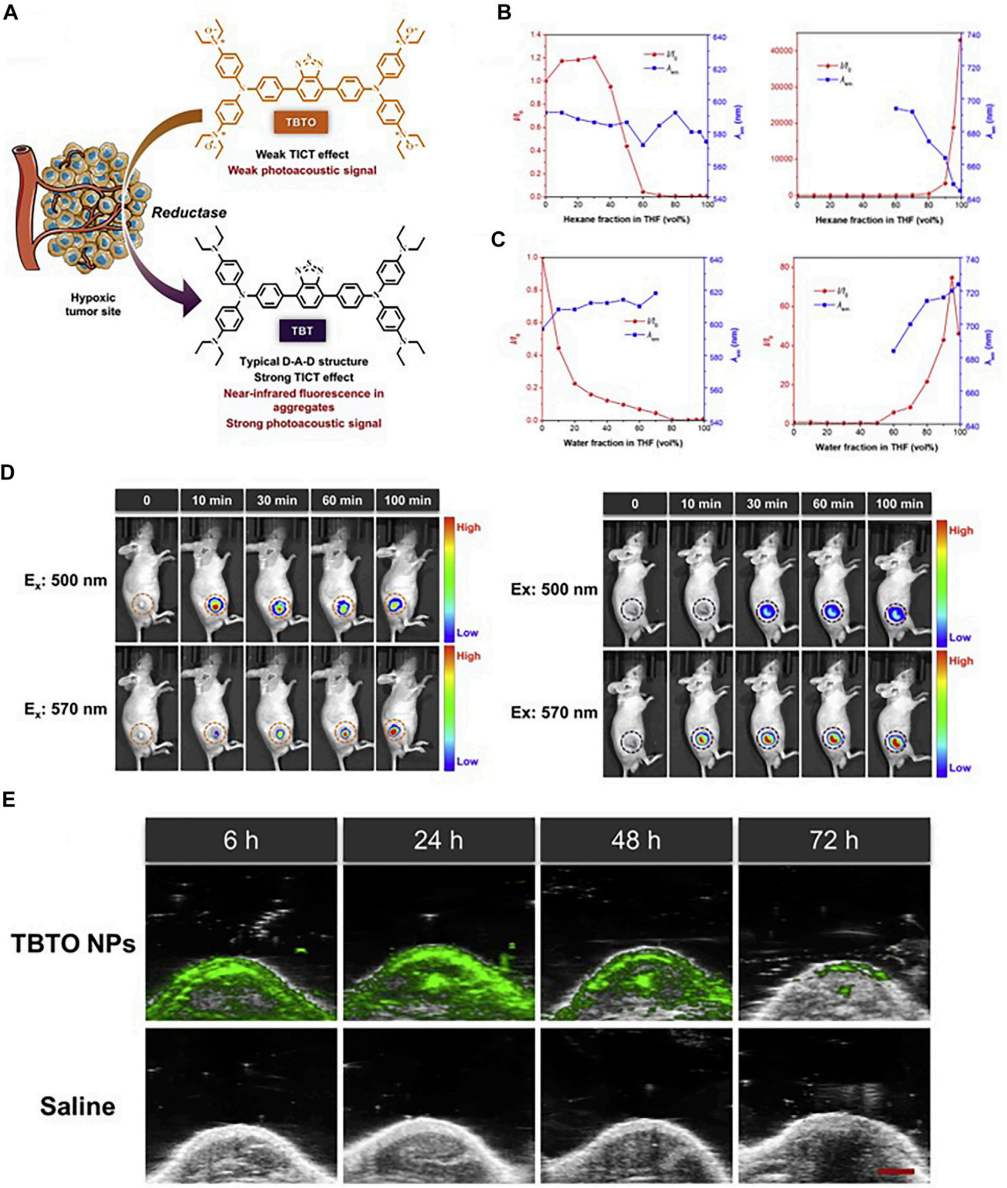
**(A)** Schematic illustration of hypoxia-activated probe TBTO. **(B)** Fluorescence emission changes of TBTO (left) and TBT (right) in THF with different fractions of hexane. **(C)** Fluorescence emission changes of TBTO (left) and TBT (right) in THF with different fractions of water. **(D)** Time-lapse FI of mice before and after intra-tumoral injection with TBTO NPs (left) and TBT NPs (right). **(E)** *In vivo* PA images of the tumor site in the mice after tail intravenous injection with TBTO NPs (200 μl, 1 mg/ml) or saline (200 μl) as the control. Reproduced with permission from Li M. et al. (2021). Copyright 2021, Cell Press.

**FIGURE 13:**
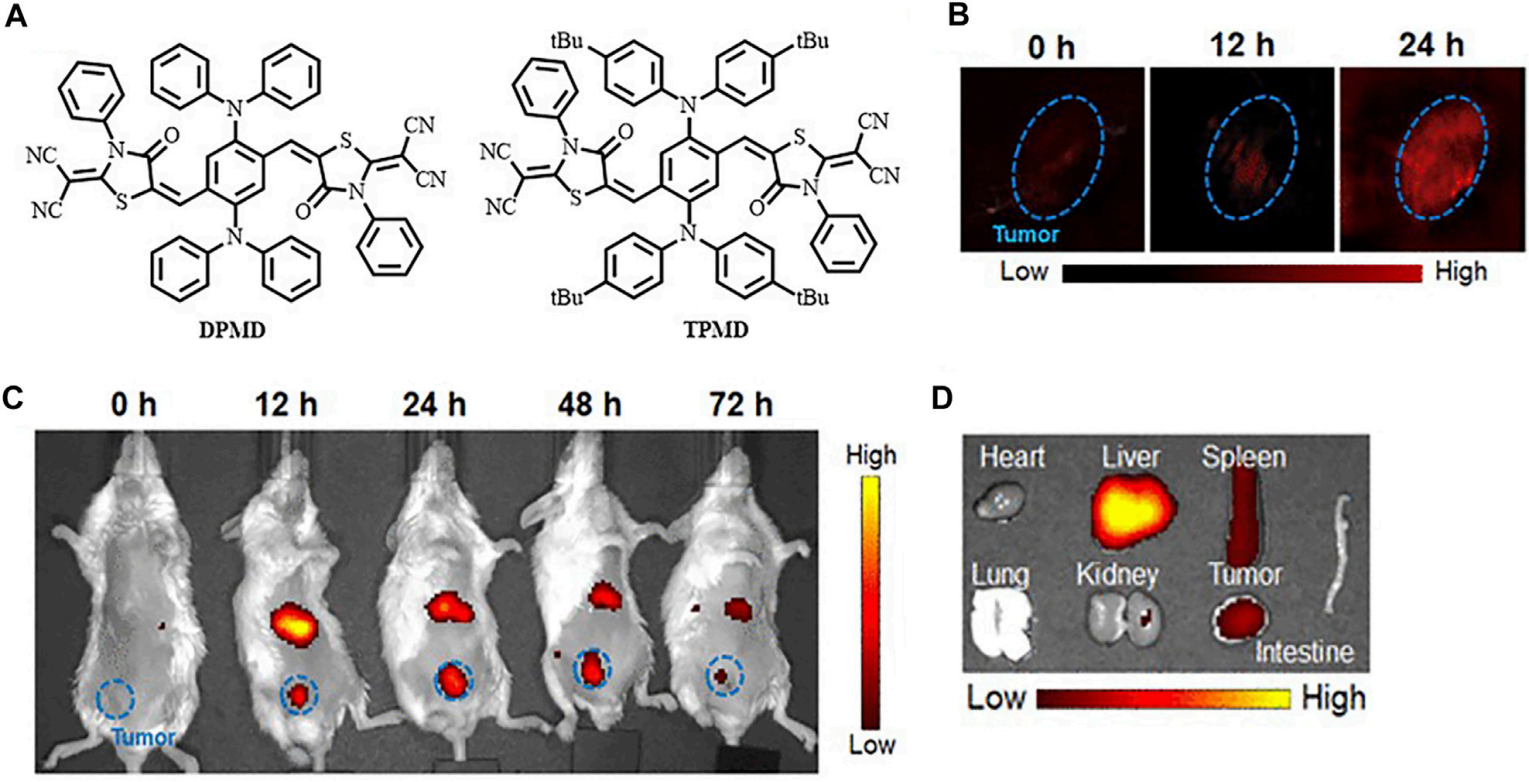
**(A)** Chemical structures of DPMD and TPMD. **(B)** *In vivo* PAI of 4T1 subcutaneous tumor-bearing mice after intravenous injection of TPMD NPs. **(C)** *In vivo* fluorescence images at different time points. **(D)** *Ex vivo* FI of the tumor and major organs from the TPMD NPs-treated mice at 24 h postinjection. Reproduced with permission from Liu et al. (2021). Copyright 2021, American Chemical Society.

In the recent 5 years, NIR-I fluorophores in the reported bimodal FI/PAI probes are often served as the CAs of PAI as well. These fluorophores, including natural origin (Zhou et al., 2017; Zheng et al., 2018; Siwawannapong et al., 2020), cyanine derivatives (Baart et al., 2021; Doan et al., 2021; Mu et al., 2021; Nishio et al., 2021), and other types of organic dyes (Park et al., 2020; Shin et al., 2021; Wang et al., 2022), had strong and broad optical absorption in the NIR-I region to provide PA signals. Apart from the broad applications of the traditional cyanine dyes, researchers have devoted much attention to developing new structures to obtain better absorption and emission properties in this field. For example, Zhang et al. had transformed the existing hemicyanine dyes with sulfur substitution to balance the energy between fluorescence and photoacoustic effects for optimized NIRF/PA dual ratiometric scaffolds. AS-Cy-NO_2_ was constructed to quantify hypoxia extent in xenograft breast cancer models through the dual ratiometric NIRF/PA imaging (Figure 11) (Zhang et al., 2022). Besides, BODIPY had also been widely applied in this field (Wang X. et al., 2019; Wu et al., 2019a; Zhang et al., 2019). Due to the deficiency of hydrophobic and water-insoluble interactions of BODIPY, the probes reported by Wang et al. and Zhang et al. were prepared with polymers or proteins to increase the solubility in aqueous media (Wang X. et al., 2019; Zhang et al., 2019). In addition, croconaine (CR) dyes, a series of organic pseudo-oxocarbon dyes prepared from 4,5-dihydroxy-4-cyclopentene-1,2,3-trione, had great application in this field with the merit of excellent thermal stability, spectral tunability, and photobleaching resistance. In order to provide obvious dual-modal FI/PAI at tumor sites, they were further modified to improve biocompatibility, target tumors, and (or) assemble into NPs (Tang et al., 2017; Yu et al., 2018; Gao et al., 2021). Nevertheless, the effect of ACQ had distinctly limited the biomedical applications of FI/PAI. In this context, the blossom of AIEgens has brought new vigor. Li and his co-workers rationally synthesized a hypoxia-activable probe TBTO which could undergo bioreduction in a hypoxic microenvironment and be converted to an AIE-active product TBT. *In vitro* and *in vivo* assessments revealed the FI/PAI ability of TBT (Figure 12) (Li M. et al., 2021). Additionally, Liu et al. designed two new AIEgens DPMD and TPMD with a cross-shaped D-A structure. Further experiments demonstrated that TPMD possessed a brighter NIR fluorescence signal for FI, more effective ROS generation for PDT, and higher photothermal effect for PAI, PTT in 4T1 tumor-bearing mice (Figure 13) (Liu et al., 2021).

As the excitation wavelength of these probes in emitting fluorescence was usually different from the absorption wavelength to produce PA signals, it was hard to offer equally high optical signals and acoustical signals at the same wavelength. Moreover, even at different wavelengths, the photoacoustic conversion would still affect its fluorescence efficiency for the same material (Wang S. et al., 2018). Under this circumstance, more efforts have been devoted to combining strong photothermal agents with typical NIR-I fluorophores in novel bimodal nanoprobes for FI/PAI in which the fluorophores were only required to offer fluorescence signals.

Gold nanomaterials have been widely used as photoacoustic CAs in the dual-modal probes of FI/PAI (Manivasagan et al., 2019; Zhan et al., 2019). For example, gold nanostars (GNSs), which have a large absorption peak in infrared light, are not only an optimal photothermal conversion material but also a potential drug carrier. Liu et al. entrapped ICG on the surface of calcium carbonate–encapsulated gold nanostars to form a pH-responsive nanoprobe GNS@CaCO_3_/ICG. It successfully achieved the integration of diagnosis and treatment, providing a distinct FI/PAI and a synergetic PTT/PDT toward tumor tissues (Liu Y. et al., 2017). Later, they reported two other GNSs-based FI/PAI probes, GNS@BSA/I-MMP_2_ NPs and GNS@IR820/DTX-CD133, in which bull serum albumin (BSA) and PEG were, respectively, used to increase the stability and drug loading efficiency of GNSs (Xia et al., 2019; Tan et al., 2020). As for gold nanorods (AuNRs), they possess higher efficient photothermal energy conversion and superior spectral bandwidth than gold nanospheres. So, Sun et al. developed Au nanorods–capped and Ce6-doped mesoporous silica nanorods (AuNRs-Ce6-MSNRs) to achieve tumor imaging and anti-tumor effects. Under the irradiation of the pulsed laser, PTT and PDT were performed with the guidance of FI of Ce6 and PAI of AuNRs (Sun et al., 2017). In addition, the synergy between AuNRs and ICG could obtain an enhanced photoacoustic signal and a stable fluorescent emission in pre-operative liver cancer diagnosis. It was proved by Guan and his co-workers through their synthesized probe Au@liposome-ICG (Guan et al., 2017).

Transition metal sulfide or oxide nanostructures have also attracted much attention as photothermal agents in FI/PAI. Due to the broad absorption peak tailing to the NIR region, the sulfides have been effective photoacoustic agents for tumor imaging. The group of Song reported a “four-in-one” theranostic nanoplatform Cy5.5-BSA-MoS_2_. Its functions of FI, PAI, PTT, and PDT were implemented by bioconjugated MoS_2_ nanosheets (Song et al., 2017). Li et al. had developed peptide functionalized MoS_2_ nanosheets MPPF constructed by a Michael addition reaction between Cy7-labeled furin substrate peptides (CCRRGGRVRR SVK-Cy7) and MoS_2_ nanosheets. In the presence of furin, the cleavage of the peptides could lead to the release of Cy7 followed by fluorescence recovery. Also, the PA signals of Cy7 at 768 nm (PA_768_) decreased rapidly and the PA signals originating from MoS_2_ nanosheets at 900 nm (PA_900_) decreased slowly, which resulted in the ratio of PA_768_ to PA_900_ as an indicator to tumor cells and tumor-bearing mice (Li X. et al., 2021). While Li et al. constructed core-satellite ICG/DOX@Gel-CuS nanomedicines (NMs) for *in vivo* real-time FI/PAI of the process of enzyme-activatable drug release. The fluorescence of ICG was initially shielded by the satellite CuS NPs in the NMs. It increased proportionate to the amount of DOX released from NMs owing to the erosion of the gelatin matrix by protease of the tumor site, whereas the photoacoustic signal from CuS NPs was not affected in this process, thus providing real-time NMs tracking (Li X. et al., 2019). Meanwhile, Sun and his co-workers reported a nanocombination PDC/P@HCuS based on CuS to actualize chemo-phototherapy of breast cancer tracking by FI/PAI (Sun Y. et al., 2019). With regard to transition metal oxide nanosystems, hydrogenated titanium dioxide (TiO_2-x_) (Guo et al., 2017) and oxygen-deficient zirconia (ZrO_2−x_) (Sun et al., 2019b) were evaluated in FI/PAI of tumors.

Additionally, polymers with easy-modification surface, including polyaniline (PANI) (Wang et al., 2016), polypyrrole (PPy) (Sun W. et al., 2019), polydopamine (PDA) (Wang J. et al., 2019), and poly(amidoamine) (PAMAM) (Lesniak et al., 2021) were common CAs to construct multifunctional nanoprobes for dual-modal FI/PAI as well. Peptides were also proved to be feasible as PA CAs in FI/PAI probes (Han et al., 2019).

## 3 Conclusion and Outlook

Multimodal imaging based on NIR-I fluorescence imaging is an ideal strategy to provide accurate information with high spatial resolution and detection sensitivity, enabling precise diagnosis and preoperative planning, as well as intraoperative tumor resection guidance. In this review, we summarize the recent progress in the field of bimodal imaging using NIR-I fluorescent dye-based probes, including FI/NMI (SPECT or PET), FI/MRI, FI/CT imaging, and FI/PAI. Remarkably, the design of nanoprobes integrating the advantages of nanomaterials and various imaging modalities shows great potential in the application of advanced *in vivo* imaging.

With the promising achievements mentioned above, the clinical translation of more FI-based bimodal probes is highly expected, but it still remains challenging. Safety is of utmost concern with all nano-related agents. Since the translation of a newly designed nanoprobe from basic research to clinical trials is largely determined by its biosafety profile, the characterizations of nanoparticles must be fully described and validated in appropriate models. Moreover, nanomaterials generally result in long-term *in vivo* retention after intravenous administration which is attributed to the reticuloendothelial system (RES). A possible solution toward the related adverse nano-biological effects is to develop surface modification methods or construct biodegradable and/or renal clearable nanoprobes. Besides, biological nanovectors such as exosomes are emerging as ideal delivery systems to help cargos escape phagocytosis of RES. The reproducibility of high-quality nanoprobes is also highly concerned. The large-scale production of nanoprobes with high quality might be difficult in practical application due to the huge influence of tiny variations in multiple properties toward the final nanoparticles. In comparison, more self-assembled probes such as novel AIEgens could be constructed without using nanomaterials as carriers by controlling their accurate molecular structure, simple molecular design, and repeatable large-quantities synthesis, providing a promising strategy to minimize the effects of these defects. Furthermore, various parameters in the tumor microenvironment could be utilized as the triggers in the design of smart nanoprobes. As most of the fluorescent dyes are either poor in water solubility or integrated into nanoparticles, it would lead to aggregation. Under this circumstance, the fluorescence might be quenched owing to the effect of ACQ. Thus, the differences between tumor and normal tissues could be a great trigger for activable probes to regain fluorescence signals *in vivo*. Additionally, multifunctional bimodal NPs with targeting groups also have a high potential for imaging-guided therapy. It is attributed to the property of nanomaterials of easily combining different functional blocks. On the one hand, the bimodal nanoprobes with the ability of cancer theranostics possess the abilities of tracing and treating. On the other hand, these nanoprobes could provide immediate feedback on the treatment outcome, enabling real-time evaluation of the prognosis. Nevertheless, to provide nice images and efficient therapy simultaneously, it is highly significant to balance the energy between radiative and non-radiative transition.

Overall, the included NIR-I fluorescence-based bimodal imaging has provided a new way for cancer diagnosis. Other FI-based bimodal techniques, such as fluorescence imaging/ultrasound and fluorescence imaging/surface-enhanced Raman spectroscopy, have shown remarkable advances as well (Jeong et al., 2015; Wang L. et al., 2019; Pal et al., 2019; Qi et al., 2019). These technologies will enable the design of theranostic probes for practical biomedical applications, thus further increasing the demands of more advanced fluorescent dyes. Meanwhile, inspired by the successful applications of FI-based bimodal probes, more efforts have been devoted to the construction of FI-related tri-or-more modality imaging probes to achieve more comprehensive diagnostic information (Carrouée et al., 2015; Persico et al., 2020; Shanmugam et al., 2021). By the rational design of a multimodality imaging platform and structural optimization of NIR-I fluorophores, we hope that fluorescence-based multimodal probes will gain more momentum to play an increasingly important role in cancer theranostics in the near future.
