# Supplementary material for: NIR-I Dye-Based Probe: A New Window for Bimodal Tumor Theranostics
Source: Front Chem. 2022 Mar 23;10:859948. doi: 10.3389/fchem.2022.859948 (PMC8984032; doi:10.3389/fchem.2022.859948)
Supplement: Supplementary file 1 [file Table1.DOCX]

Supplementary Material

# Supplementary Table

Supplementary Table 1. The details of the involved FI/MRI probes.

Note: a: fluorescent-labelling magnetic nanoparticles; a1: Gd-labelling fluorescent nanoparticles; b: integrating NIR-I fluorophores and CAs into the nanoscale matrix; c: self-assembly.

|  | Fluorophore | CAs for MRI | Nanoplatforms | Methods of constructing FI/MRI nanoprobes | Probes | Hydrodynamic size of nanoprobes (nm) | Zeta potential (mV) | λ_em_  (nm) | Relaxivity (mM ^-1^ s^-1^) | Application | Reference |
| --- | --- | --- | --- | --- | --- | --- | --- | --- | --- | --- | --- |
| 1 | Cy7 | *T*_1_-weighted Gd-DOTA | - | not nanoprobe | **Gd-Cy7-PTP/RGD** | not nanoprobe | not nanoprobe | 748 | r_1_ 3.57  (7.0 T) | FI/MRI of pancreatic cancer | (Wang et al., 2018) |
| 2 | Cy5.5 | *T*_2_-weighted dextran-coated iron oxide nanoparticles | Dextran | a | **MN-EPPT** | - | - | ~ 695 | - | FI/MRI of uMUC1-positive pancreatic cancer | (Wang et al., 2016) |
| 3 | CyTE777 | *T*_2_-weighted a manganese-doped iron oxide nanoparticle core | - | a | **MnMEIO-CyTE777-(Bis)-mPEG** | 42.5 ± 1.8 | - 3.8 ± 0.3 | 820 | r_2_ 229.8 ± 3.9  (7.0 T) | FI/MRI of tumors expressing both  HER2/neu and/or EGFR | (Wu et al., 2016b) |
| 4 | ICG | *T*_2_-weighted Fe/FeO nanocrystals | - | a | **DOX-ICG@Fe/Feo-PPP** | 218.9 ± 52.1 | - | 810 | r_2_ 130.7  (3 T) | FI/MRI, chemotherapy (DOX), PTT (ICG), and PDT (ICG) of KB tumor-bearing mice | (Wang et al., 2019) |
| 5 | Cy5.5 | *T*_2_-weighted iron oxide NPs | Dextran | a | **MN-EPPT** | - | - | ~ 695 | - | FI/MRI of tumors expressing uMUC1 | (Zhao et al., 2019) |
| 6 | cypate | *T*_2_-weighted iron oxide NPs | Red blood cell membrane | a | **Cyp-MNC@RBC** | - | - | 845 | - | FI/MRI and PTT (cypate) of tumor | (Wang et al., 2020b) |
| 7 | hepthamethine cyanine (HMC) | *T*_2_-weighted ferumoxytol (FMX) | Carboxymethyl dextran | a | **HMC-FMX** | 37.0 ± 3.0 | −11.8 ± 0.3 | 799 | r_2_ 98.7 ± 9.5  (3.0 T) | FI/MRI, or chemotherapy (paclitaxel or cisplatin) of glioblastoma multiforme tumor | (Reichel et al., 2020) |
| 8 | IR820 | *T*_2_-weighted SPIOs | PCLA-PEG-PCLA polymers | a | **NC-SPIOs-IR820-PTX** | ~ 212 | - | ~820 | r_2_ 216.17  (7.0 T)  (**NC-SPIOs**) | FI/MRI, chemotherapy (PTX), and PTT (IR820) of 4T1 cancer bearing Balb/C mice | (Liao et al., 2017) |
| 9 | ICG | *T*_2_-weighted SPIOs | Liposome | a | **SPIO@Liposome-ICG-RGD** | 126 ± 21 | - | ~810 | r_2_ 363.4  (1.0 T) | FI/MRI of primary liver tumors | (Chen et al., 2017) |
| 10 | Cy5.5 | *T*_2_-weighted SPIOs | Silica shell | a | **NF-SIONs** | 37.84 | - | 691 | r_2_ 50.36  (9.4 T) | FI/MRI of glioblastoma multiforme | (Lee et al., 2018) |
| 11 | Cy5 | *T*_2_-weighted SPIOs | Micelle | a | **SPIO/DSPE-PEG5k-(BOM&Cy5)** | 145±56 | −2.9±1.1 | - | r_2_ 493.9  (3.0 T) | FI/MRI of breast cancer | (Li et al., 2019a) |
| 12 | IR-783 | *T*_2_-weighted Fe_3_O_4_ NPs | - | a | **GPC@IR783-Fe_3_O_4_** | 83.2 | ~-12 | 790 | - | FI/MRI of Huh-7 tumor-bearing mice | (Liang et al., 2017) |
| 13 | Cy5.5 | *T*_2_-weighted Fe_3_O_4_ | Poly(succinimide) graft copolymer | a | **CMNPs-Cy5.5-fol** | 44.7±15.4 | − 29.3 ± 2.3 | ~ 695 | r_2_ 181  (4.7 T) | FI/MRI of tumors overexpressing folate receptor | (Yang et al., 2018a) |
| 14 | Ce6 | *T*_2_-weighted Fe_3_O_4_ NPs | - | a | **Fe_3_O_4_-PEG-G5-MMP2@Ce6** | 105.44 | +32.44 | 700 | r_2_ 165.24  (0.5 T) | FI/MRI and PDT (Ce6) of MGC-803 tumor bearing mice | (Duan et al., 2019) |
| 15 | IR820 | *T*_2_-weighted Fe_3_O_4_ NPs | - | a | **Fe@IR820** | 110 | -17.3 | 840 | r_2_ 125.6  (-) | FI/MRI, PDT (IR820), and PTT (IR820) of hepatocellular carcinoma tumor | (Xie et al., 2020) |
| 16 | ICG | *T*_2_-weighted Fe_3_O_4_@CS nanospheres | Chitosan (CS) | a | **Fe_3_O_4_@CS-ICG/DOX** | ∼189 | - | ~ 835 | r_2_ 139.3  (0.55T)  (**Fe_3_O_4_@CS nanospheres**) | FI/MRI, chemotherapy (DOX), and PTT (ICG) of HeLa tumor bearing mice | (Gao et al., 2016) |
| 17 | Cy5.5-labeled HSA | *T*_2_-weighted self-assembly of Fe_3_O_4_ NPs and redox-responsive polymer ligands (termed **RMNs**) | - | a | **RMNs-HSA-Cy5.5** | 60−70 (**RMNs**) | +19 (**RMNs**) | 707−715 | r_2_ 73.16  (7.0 T)  (**RMNs**) | FI/MRI of breast cancer | (Yang et al., 2017) |
| 18 | Ce6 | *T*_2_-weighted Fe_3_O_4_ nanoparticles and *T*_1_-weighted MnO_2_ nanoparticles | Poly(acrylic acid) (PAA) | a | **MUCNPs@BPNs-Ce6** | - | - | 660 | r_2_ 5.71  and r_1_ 9.79  (3.0 T) | FI/MRI, PDT (black phosphorus and Ce6), and PTT (black phosphorus) of HeLa tumor bearing nude mice | (Zhang et al., 2020) |
| 19 | ICG | *T*_1_-weighted prussian blue nanoparticles | Bovine serum albumin (BSA) | a | **PB-BSA-ICG** | 33 ± 5 | −17 ± 5 | - | r_1_ ~1.33  (3 T) | FI/MRI, PDT (ICG), and PTT (ICG) of tumor | (Sahu et al., 2016) |
| 20 | ICG | *T*_1_-weighted SF@MnO_2_ NPs | Regenerated silk fibroin (SF) | a | **SMID** | 139.6 | ~−17 | - | r_1_ 11.65  (3.0 T) | FI/MRI, chemotherapy (DOX), PTT (ICG), and PDT (ICG) of 4T1 tumor-bearing mice | (Yang et al., 2019) |
| 21 | Cy7 | *T*_1_-weighted manganese oxide–mesoporous silica nanoparticles (Mn-Msns) | Mesoporous silica | a | **PSA-Mn-Msn-Cy7** | 85.4 ± 14.8 | - | 773 | - | FI/MRI of prostate cancer | (Du et al., 2020) |
| 22 | Cy7.5 | *T*_1_-weighted Gd_2_O_3_/CuS hybrid nanodots | Bovine serum albumin (BSA) | a | **Cy7.5-Gd_2_O_3_/CuS NDs** | 18.2 ± 2.2  (**Gd_2_O_3_/CuS NDs**) | - | - | r_1_ 27.2  (1.5 T) | FI/MRI and PTT of 4T1 tumor bearing mice | (Wen et al., 2017) |
| 23 | IRDye800 | *T*_1_-weighted Gd_2_O_3_ | Bovine serum albumin (BSA) | a | **RVG&IRDye800-Gd_2_O_3_TNs** | - | - | 806 | r_1_ 9.90  (1.0 T) | FI/MRI of neuroblastoma | (Jin et al., 2019) |
| 24 | ICG | *T*_1_-weighted Gd_2_O_3_ | Human serum albumin (HSA) | a | **PIGH NPs** | 115 | −10.9 | - | r_1_ 13.75  (1.5 T) | FI/MRI, chemotherapy (PTX), and PTT (ICG) of 4T1 tumor- bearing mice | (Hao et al., 2019) |
| 25 | NIR783 | *T*_1_-weighted gadofullerences (GdF) | Carbon cage | a | **GdF-SS-NIR783** | 145 | −20.87 | 810 (with GSH) | r_1_ 6.92  (7.0 T) | FI/MRI of HeLa tumor-bearing mice | (Li et al., 2018a) |
| 26 | ICG | *T*_1_-weighted Gd^3+^-doped MLDH nanosheets | Monolayered-double-hydroxide (MLDH) nanosheets | a | **DOX&ICG/**  **MLDH** | ~82 | −12.2 | - | r_1_ 7.93  (0.5 T) | FI/MRI, chemotherapy (DOX), PTT (ICG), and PDT (ICG) of HepG2 tumor bearing nude mice | (Peng et al., 2018) |
| 27 | Cy5.5 | *T*_1_-weighted Gd-doping CuS micellar NPs | CuS micelle | a | **T-MAN** | ~61 | −15.2 | 690 (with MMP-2) | r_1_ 60.0±1.7  (1 T) | FI/MRI and PTT of subcutaneous and metastatic gastric tumors. | (Shi et al., 2019) |
| 28 | ICG | *T*_1_-weighted Gd^3+^-decorated CMs | Casein micelles (CM) | a | **CM-Gd_7_-J-ICG** | 166 | - | - | r_1_ 4.72  (1.4 T) | FI/MRI and PTT (ICG) of tumor | (Picchio et al., 2021) |
| 29 | Ce6 | *T*_1_-weighted Gd^3+^ | Poly(lactic-co-glycolic acid) nanoparticles (PLGA NPs) | a1 | **HAGCP-NPs** | ~200 | 58.07 | 674 | r_1_ 19.59  (4.7 T) | FI/MRI and PDT (Ce6) of A549 tumor-bearing mice | (Park et al., 2017) |
| 30 | Ce6 | *T*_1_-weighted Gd^3+^ | Self-assembling of the functionalized 1,2-distearoyl-sn-glycero-3phosphoethanolamine-poly(ethylene glycol)-2000 | a1 | **DSPM@Ce6@Gd NPs** | ~157.1 | - | 660 | 42.0  (0.5 T) | FI/MRI and PDT (Ce6) of A549 tumor-bearing mice | (Yang et al., 2022) |
| 31 | Purpurin 18 | *T*_1_-weighted Mn^2+^ | Nanogels | a1 | **DPH-Mn NGs** | 66.95 ± 1.93  (**DPH NGs**) | ~−10  (**DPH NGs**) | 710 | r_1_  (7 T) | FI/MRI, chemotherapy (10- hydroxycamplothecin), and PDT (purpurin 18) of 4T1 tumor-bearing mice | (Ma et al., 2021) |
| 32 | Cy5.5 | *T*_1_-weighted Gd-DTPA | Holo-Transferrin (Tf) | b | **Cy5.5-Tf-Gd-DTPA** | - | - | 692 | r_1_ 4.21  (7.0 T) | FI/MRI of mice with subcutaneous H1299 xenografte tumors | (Cai et al., 2016) |
| 33 | Cy5.5 | *T*_1_-weighted Gd^3+^-DTPA | Dendron-grafted polylysine (DGL) | b | **DGL-U11** | 22.98 | 6.3 | ~690 | r_1_ 6.2 /Gd^3+^  (7.0 T) | FI/MRI of precancerous pancreatic intraepithelial neoplasia (PanIN) tissues and pancreatic ductal adenocarcinoma lesions | (Li et al., 2018b) |
| 34 | maleimide derivative cyanine dye (Mal-Cy5.5) | *T*_1_-weighted Gd^3+^-DTPA | Mesoporous organosilica nanoparticles (MONs) | b | **MON-Gd-Cy5.5-RGD** | 80 ± 10 (**MONs**) | 22 | 707 | r_1_ 2.85  (3.0 T) | FI/MRI of tumor | (Li et al., 2019b) |
| 35 | Cy7 | *T*_1_-weighted Gd-DOTA | Gold nanocages (AuNCs) | b | **ORI-GPC1-NPs** | 88 | −10.2 | 767 | r_1_ 16.33  (3.0 T) | FI/MRI of pancreatic tumor xenografted mice | (Qiu et al., 2018) |
| 36 | IRDye800CW | *T*_1_-weighted Gd-DOTA | Liposomal cerasome NPs | b | **PD-L1-PCI-Gd** | 100.4 ± 5.5 | −41.18 ± 3.52 | ~800 | r_1_ ~12.00  (7.0 T) | FI/MRI and chemotherapy (PTX) of xenografted 4T1 breast tumor and CT26 colon tumor | (Du et al., 2018) |
| 37 | Cy5.5 | *T*_1_-weighted Gd^3+^-DOTA | Physalis mottle virus nanoparticles (PhMV NPs) | b | **Gd-Cy5.5-PhMV-DGEA** | ∼35.7 | - | - | r_1_ 31.0 (1.5T) / 8.2 (7T) (**Gd-Cy5.5-PhMV-mPEG**) | FI/MRI of prostate cancer | (Hu et al., 2019) |
| 38 | Cy7 | *T*_1_-weighted Gd^3+^-DOTA | Gold nanocarrier | b | **GPC1-GEM-NPs** | ~89 | -10.4 | - | r_1_ 18.308  (3.0 T) | FI/MRI and chemotherapy (gemcitabine) of pancreatic cancer | (Qiu et al., 2019) |
| 39 | Dylight800 | *T*_1_-weighted Gd^3+^-DOTA | DNA bipyramid nanostructure | b | **T-Pc-Bp-Gd** | 33.4 ± 0.3 | 0 | - | r_1_ 7  (7.0 T) | FI/MRI of tumor | (Song et al., 2020) |
| 40 | CF750 | *T*_1_-weighted gadolinium lipid | thermosensitive liposome | b | **iTSL-S_M_/C_C_** | 142 ± 5.0 | -6.5±2.4 | ~750 | - | FI/MRI and chemotherapy (SN-38 and carboplatin) of triple negative breast cancer xenografts tumors | (Cressey et al., 2021) |
| 41 | Cy7 | *T*_1_-weighted Fe^3+^-DOTA | Au@SiO2@Au | b | **FeCy7−NM** | - | - | 761 | r_1_ 5.9  (4.7 T) | FI/MRI of tissues | (Henderson et al., 2018) |
| 42 | Cy 5.5 | *T*_2_-weighted ferrimagnetic nanocubes | Glycol chitosan NPs | b | **pMCNP** | 481.8±8.7 | 32.3±0.9 | 695 | r_2_ 180  (3.0 T) | FI/MRI of bladder cancer | (Key et al., 2016) |
| 43 | Ce6 | *T*_2_-weighted Fe_3_O_4_ NPs | Dextran | b | **DSSCe6@Fe_3_O_4_** | 198 | −9.19 | ~650 (with dithio-threitol) | r_2_ 194.4  (3 T) | FI/MRI and PDT (Ce6) of tumor | (Ding et al., 2017) |
| 44 | triphenylamine divinylanthracene-dicyano (TAC) | *T*_2_-weighted Fe_3_O_4_ NPs | Poly(L-lactic-co-glycolic acid) nanoparticles | b | **anti-VEGF/OA-Fe_3_O_4_/TAC@PLGA NPs** | - | -30 | 670 | r_2_ 86.2  (-) | FI/MRI and PTT (Fe_3_O_4_) of tumor | (Ma et al., 2019) |
| 45 | Cy5.5 | *T*_2_-weighted oleic acid-coated iron oxide NPs | Thermo-sensitive magneto liposomes | b | **MTX-MagTSLs** | 107.5 ± 1.19 | 16.8±1.2 | - | r_2_ 60.06  (7 T) | FI/MRI and chemotherapy (DOX) of HeLa tumor-bearing nude mice | (Guo et al., 2018) |
| 46 | MHI-148 | *T*_2_-weighted SPIOs | Chitosan | b | **MMGCPTs** | 254 | 267 | 774 | r_2_ 40  (3 T) | FI/MRI, chemotherapy (PTX), and PTT (MHI-148) of CT26 tumor | (Sasikala et al., 2018) |
| 47 | Cy7.5 | *T*_2_-weighted dysprosium (Dy^3+^) | Tobacco mosaic virus (TMV) | b | **Dy-Cy7.5-TMV-DGEA** | - | - | - | r_2_ 326 (7T) / 399 (9.4T) | FI/MRI of PC-3 prostate cancer | (Hu et al., 2017) |
| 48 | ICG | *T* _1_-weighted Gd-DOTA | - | c (depend on folic acid (FA) modified PEI-PEG-gadoteric acid) | **ICG-FA-PPD** | 211.9 ± 1.1 | 25.5 ± 0.7 | 812 | r_1_ 9.43 × 10^-3^  (0.5 T) | FI/MRI, PTT (ICG), and PDT(ICG) of U-87 MG tumor | (Wu et al., 2016a) |
| 49 | IR780 | *T*_1_-weighted Gd-DOTA | - | c (depend on pentapeptide CREKA (Cys-Arg-Glu-Lys-Ala) (DSPE-PEG-CREKA)) | [**DPC@ICD-Gd-Tic**](mailto:DPC@ICD-Gd-Tic) | 127±12.3 (**DPC@ICD-Gd**) | -34.74±5.87 (**DPC@ICD-Gd**) | ~800 (**DPC@**  **ICD-Gd**) | r_1_ 3.052  (-) | FI/MRI, chemotherapy (Tic), and PTT (IR780) of 4T1 tumor | (Zhang et al., 2021) |
| 50 | amantadine-modified Cy7 | *T*_1_-weighted amantadine-modified Gd-DOTA | - | c (depend on hyaluronic acid–βcyclodextrin (HA–CD)) | **HA–CD–GC NPs** | ~32 | -10 | - | r_1_ 11.4  (0.5 T) | FI/MRI of tumor | (Wang et al., 2017) |
| 51 | Ce6 | *T*_1_-weighted Mn^2+^ | - | c (depend on human serum albumin) | **HSA-MnO_2_-Ce6 NPs** | 118.6±8.1 | -24.8±1.0 | - | r_1_ 1.85  (7.0 T) | FI/MRI and PDT (Ce6) of bladder tumor | (Lin et al., 2018) |
| 52 | IR780 | *T*_1_-weighted Mn^2+^ | - | c (depend on PCL-b-PIEtMn and PCL-b-PEG) | **DOX&IR-780@NPs** | ~150 | - | - | r_1_ 7.05  (3.0 T) | FI/MRI, chemotherapy (DOX), and PTT (IR780) of 4T1 tumor | (Gao et al., 2020) |
| 53 | PIMA-PEG-Dopa-Cyst-Ce6 | *T*_2_-weighted SPIONs | - | c (depend on PIMA and PEG) | **RMNs** | ~90 | -18.2 ± 0.6 | 672 | r_2_ 3.0  (3.0 T) | FI/MRI and PDT (Ce6) of breast tumor | (Yang et al., 2018b) |
| 54 | 2-(4-bromophenyl)-3-(4-(4-(diphenylamino)styryl)phenyl)fumaronitrile (TB) | *T*_2_-weighted SPIO | - | c (depend on PS-PEG) | **TSP NPs** | ~100 | 29.1±0.7 | 655 | r_2_ 51.82  (1.5 T) | FI/MRI of liver tumor | (Meng et al., 2019) |
| 55 | IR780 | *T*_2_-weighted SPIO | - | c (depend on hexahydrophthalic anhydride modified stearic acid-graftedpolyethylenimine (SPA)) | **SP-SPIO-IR780 and SPA-SPIO-IR780** | 44.97±10.12 (**SP-SPIO-IR780**) 53.01±9.54 (**SPA-SPIO-IR780**) | 31.9 (**SP-SPIO-IR780**)  25.5 (**SPA-SPIO-IR780**) | 845 | r_2_ 287.82 (**SP-SPIO-IR780**)  254.09 (**SPA-SPIO-IR780**)  (1.5 T) | FI/MRI, PTT (IR780), and PDT(IR780) of triple-negative 4T1 breast tumor | (Wang et al., 2020a) |
| 56 | pheophorbide A (Pa) | *T* _1_-weighted Mn^2+^ | - | c | **PaIr NPs** | 88 | 30.9 | ~700 | r_1_ 4.38  (7 T) | FI/MRI, chemotherapy (irinotecan), PTT (Pa), and PDT(Pa) of tumor | (Xue et al., 2018b) |
| 57 | pheophorbide A (Pa) | *T* _1_-weighted Mn^2+^ | - | c | **pPhD NPs** | ~79 | ~12 | ~700 | r_1_ 2.89  (7 T) | FI/MRI, chemotherapy (DOX), PTT (Pa), and PDT(Pa) of tumor | (Xue et al., 2018a) |
| 58 | merocyanine, Cy-Cl | *T*_1_-weighted Gd-DOTA | - | c (under ALP) | **P-CyFF-Gd** | ∼66 | - | 710 (**CyFF-Gd**) | r_1_ 20.1 ± 0.5 (**P-CyFF-Gd**+ALP)  (0.5 T) | FI/MRI of liver tumor | (Yan et al., 2019) |

Reference

Cai, J., Gu, B., Cao, F., and Liu, S. (2016). A transferrin-target magnetic/fluorescent dual-mode probe significantly enhances the diagnosis of non-small cell lung cancer. *Oncotarget* 7**,** 40047-40059. doi:10.18632/oncotarget.9482

Chen, Q., Shang, W., Zeng, C., Wang, K., Liang, X., Chi, C., et al. (2017). Theranostic imaging of liver cancer using targeted optical/MRI dual-modal probes. *Oncotarget* 8**,** 32741-32751. doi:10.18632/oncotarget.15642

Cressey, P., Amrahli, M., So, P., Gedroyc, W., Wright, M., and Thanou, M. (2021). Image-guided thermosensitive liposomes for focused ultrasound enhanced co-delivery of carboplatin and SN-38 against triple negative breast cancer in mice. *Biomaterials* 271**,** 120758. doi:10.1016/j.biomaterials.2021.120758

Ding, Z., Liu, P., Hu, D., Sheng, Z., Yi, H., Gao, G., et al. (2017). Redox-responsive dextran based theranostic nanoparticles for near-infrared/magnetic resonance imaging and magnetically targeted photodynamic therapy. *Biomater. Sci.* 5**,** 762-771. doi:10.1039/c6bm00846a

Du, D., Fu, H., Ren, W., Li, X., and Guo, L. (2020). PSA targeted dual-modality manganese oxide-mesoporous silica nanoparticles for prostate cancer imaging. *Biomed. Pharmacother.* 121**,** 109614. doi:10.1016/j.biopha.2019.109614

Du, Y., Liang, X., Li, Y., Sun, T., Xue, H., Jin, Z., et al. (2018). Liposomal nanohybrid cerasomes targeted to PD-L1 enable dual-modality imaging and improve antitumor treatments. *Cancer Lett.* 414**,** 230-238. doi:10.1016/j.canlet.2017.11.019

Duan, M., Xia, F., Li, T., Shapter, J.G., Yang, S., Li, Y., et al. (2019). Matrix metalloproteinase-2-targeted superparamagnetic Fe_3_O_4_-PEG-G5-MMP2@Ce6 nanoprobes for dual-mode imaging and photodynamic therapy. *Nanoscale* 11**,** 18426-18435. doi:10.1039/C9NR06774D

Gao, Z., Liu, X., Wang, Y., Deng, G., Zhou, F., Wang, Q., et al. (2016). Facile one-pot synthesis of Fe_3_O_4_@chitosan nano-spheres for MRI and fluorescence imaging guided chemo-photothermal combinational cancer therapy. *Dalton Trans.* 45**,** 19519-19528. doi:10.1039/c6dt03897b

Gao, Z., Mu, W., Tian, Y., Su, Y., Sun, H., Zhang, G., et al. (2020). Self-assembly of paramagnetic amphiphilic copolymers for synergistic therapy. *J. Mater. Chem. B* 8**,** 6866-6876. doi:10.1039/D0TB00405G

Guo, Y., Zhang, Y., Ma, J., Li, Q., Li, Y., Zhou, X., et al. (2018). Light/magnetic hyperthermia triggered drug released from multi-functional thermo-sensitive magnetoliposomes for precise cancer synergetic theranostics. *J. Control. Release* 272**,** 145-158. doi:10.1016/j.jconrel.2017.04.028

Hao, T., Chen, Q., Qi, Y., Sun, P., Chen, D., Jiang, W., et al. (2019). Biomineralized Gd_2_O_3_@HSA nanoparticles as a versatile platform for dual-modal imaging and chemo-phototherapy-synergized tumor ablation. *Adv. Healthcare Mater.* 8**,** 1901005. doi:10.1002/adhm.201901005

Henderson, L., Neumann, O., Kaffes, C., Zhang, R., Marangoni, V., Ravoori, M.K., et al. (2018). Routes to potentially safer T-1 magnetic resonance imaging contrast in a compact plasmonic nanoparticle with enhanced fluorescence. *ACS Nano* 12**,** 8214-8223. doi:10.1021/acsnano.8b03368

Hu, H., Masarapu, H., Gu, Y., Zhang, Y., Yu, X., and Steinmetz, N.F. (2019). Physalis mottle virus-like nanoparticles for targeted cancer imaging. *ACS Appl. Mater. Interfaces* 11**,** 18213-18223. doi:10.1021/acsami.9b03956

Hu, H., Zhang, Y., Shukla, S., Gu, Y., Yu, X., and Steinmetz, N.F. (2017). Dysprosium-modified tobacco mosaic virus nanoparticles for ultra-high-field magnetic resonance and near-infrared fluorescence imaging of prostate cancer. *ACS Nano* 11**,** 9249-9258. doi:10.1021/acsnano.7b04472

Jin, Y., Li, Y., Yang, X., and Tian, J. (2019). Neuroblastoma-targeting triangular gadolinium oxide nanoplates for precise excision of cancer. *Acta Biomater.* 87**,** 223-234. doi:10.1016/j.actbio.2019.01.042

Key, J., Dhawan, D., Cooper, C.L., Knapp, D.W., Kim, K., Kwon, I.C., et al. (2016). Multicomponent, peptide-targeted glycol chitosan nanoparticles containing ferrimagnetic iron oxide nanocubes for bladder cancer multimodal imaging. *Int. J. Nanomed.* 11**,** 4141-4155. doi:10.2147/ijn.s109494

Lee, C., Kim, G.R., Yoon, J., Kim, S.E., Yoo, J.S., and Piao, Y. (2018). In vivo delineation of glioblastoma by targeting tumor-associated macrophages with near-infrared fluorescent silica coated iron oxide nanoparticles in orthotopic xenografts for surgical guidance. *Sci. Rep.* 8**,** 11122. doi:10.1038/s41598-018-29424-4

Li, C., Huang, H., Cui, R., Li, J., Guo, X., Yao, H., et al. (2018a). Fluorescent activatable gadofullerene nanoprobes as NIR-MR dual-modal in vivo imaging contrast agent. *Colloids Surf. B* 171**,** 159-166. doi:10.1016/j.colsurfb.2018.07.026

Li, H., Wang, P., Gong, W., Wang, Q., Zhou, J., Zhu, W., et al. (2018b). Dendron-grafted polylysine-based dual-modal nanoprobe for ultra-early diagnosis of pancreatic precancerosis via targeting a urokinase-type plasminogen activator receptor. *Adv. Healthcare Mater.* 7**,** 1700912. doi:10.1002/adhm.201700912

Li, L., Wu, C., Pan, L., Li, X., Kuang, A., Cai, H., et al. (2019a). Bombesin-functionalized superparamagnetic iron oxide nanoparticles for dual-modality MR/NIRFI in mouse models of breast cancer. *Int. J. Nanomed.* 14**,** 6721-6732. doi:10.2147/IJN.S211476

Li, Y., Guo, W., Su, X., Lu, N., Wu, G., Lin, O., et al. (2019b). Facile preparation of near-infrared fluorescence and magnetic resonance dual-modality imaging probes based on mesoporous organosilica nanoparticles. *J. Colloid Interface Sci.* 539**,** 277-286. doi:10.1016/j.jcis.2018.12.067

Liang, J., Zhang, X., Miao, Y., Li, J., and Gan, Y. (2017). Lipid-coated iron oxide nanoparticles for dual-modal imaging of hepatocellular carcinoma. *Int. J. Nanomed.* 12**,** 2033—2044. doi:10.2147/ijn.s128525

Liao, J., Wei, X., Ran, B., Peng, J., Qu, Y., and Qian, Z. (2017). Polymer hybrid magnetic nanocapsules encapsulating IR820 and PTX for external magnetic field-guided tumor targeting and multifunctional theranostics. *Nanoscale* 9**,** 2479-2491. doi:10.1039/c7nr00033b

Lin, T., Zhao, X., Zhao, S., Yu, H., Cao, W., Chen, W., et al. (2018). O_2_-Generating MnO_2_ nanoparticles for enhanced photodynamic therapy of bladder cancer by ameliorating hypoxia. *Theranostics* 8**,** 990-1004. doi:10.7150/thno.22465

Ma, K., Liu, G., Yan, L., Wen, S., Xu, B., Tian, W., et al. (2019). AIEgen based poly(L-lactic-co-glycolic acid) magnetic nanoparticles to localize cytokine VEGF for early cancer diagnosis and photothermal therapy. *Nanomedicine* 14**,** 1191-1201. doi:10.2217/nnm-2018-0467

Ma, X., Zhang, T., Qiu, W., Liang, M., Gao, Y., Xue, P., et al. (2021). Bioresponsive prodrug nanogel-based polycondensate strategy deepens tumor penetration and potentiates oxidative stress. *Chem. Eng. J.* 420**,** 127657. doi:10.1016/j.cej.2020.127657

Meng, L., Ma, X., Jiang, S., Ji, G., Han, W., Xu, B., et al. (2019). High-efficiency fluorescent and magnetic multimodal probe for long-term monitoring and deep penetration imaging of tumors. *J. Mater. Chem. B* 7**,** 5345-5351. doi:10.1039/c9tb00638a

Park, K.E., Noh, Y.W., Kim, A., and Lim, Y.T. (2017). Hyaluronic acid-coated nanoparticles for targeted photodynamic therapy of cancer guided by near-infrared and MR imaging. *Carbohydr. Polym.* 157**,** 476-483. doi:10.1016/j.carbpol.2016.10.015

Peng, L., Mei, X., He, J., Xu, J., Zhang, W., Liang, R., et al. (2018). Monolayer nanosheets with an extremely high drug loading toward controlled delivery and cancer theranostics. *Adv. Mater.* 30**,** 1707389. doi:10.1002/adma.201707389

Picchio, M.L., Bergueiro, J., Wedepohl, S., Minari, R.J., Alvarez Igarzabal, C.I., Gugliotta, L.M., et al. (2021). Exploiting cyanine dye J-aggregates/monomer equilibrium in hydrophobic protein pockets for efficient multi-step phototherapy: An innovative concept for smart nanotheranostics. *Nanoscale* 13**,** 8909-8921. doi:10.1039/D0NR09058A

Qiu, W., Chen, R., Chen, X., Zhang, H., Song, L., Cup, W., et al. (2018). Oridonin-loaded and GPC1-targeted gold nanoparticles for multimodal imaging and therapy in pancreatic cancer. *Int. J. Nanomed.* 13**,** 6809-6827. doi:10.2147/ijn.s177993

Qiu, W., Zhang, H., Chen, X., Song, L., Cui, W., Ren, S., et al. (2019). A GPC1-targeted and gemcitabine-loaded biocompatible nanoplatform for pancreatic cancer multimodal imaging and therapy. *Nanomedicine* 14**,** 2339-2353. doi:10.2217/nnm-2019-0063

Reichel, D., Sagong, B., Teh, J., Zhang, Y., Wagner, S., Wang, H., et al. (2020). Near infrared fluorescent nanoplatform for targeted intraoperative resection and chemotherapeutic treatment of glioblastoma. *ACS Nano* 14**,** 8392-8408. doi:10.1021/acsnano.0c02509

Sahu, A., Lee, J.H., Lee, H.G., Jeong, Y.Y., and Tae, G. (2016). Prussian blue/serum albumin/indocyanine green as a multifunctional nanotheranostic agent for bimodal imaging guided laser mediated combinatorial phototherapy. *J. Control. Release* 236**,** 90-99. doi:10.1016/j.jconrel.2016.06.031

Sasikala, A.R.K., Unnithan, A.R., Thomas, R.G., Batgerel, T., Jeong, Y.Y., Park, C.H., et al. (2018). Hexa-functional tumour-seeking nano voyagers and annihilators for synergistic cancer theranostic applications. *Nanoscale* 10**,** 19568-19578. doi:10.1039/C8NR06116E

Shi, H., Sun, Y., Yan, R., Liu, S., Zhu, L., Liu, S., et al. (2019). Magnetic semiconductor Gd-doping CuS nanoparticles as activatable nanoprobes for bimodal imaging and targeted photothermal therapy of gastric tumors. *Nano Lett.* 19**,** 937-947. doi:10.1021/acs.nanolett.8b04179

Song, L., Wang, Z., Liu, J., Wang, T., Jiang, Q., and Ding, B. (2020). Tumor-targeted DNA bipyramid for in vivo dual-modality imaging. *ACS Appl. Bio Mater.* 3**,** 2854-2860. doi:10.1021/acsabm.9b01096

Wang, H., Sun, D., Liao, H., Wang, Y., Zhao, S., Zhang, Y., et al. (2017). Synthesis and characterization of a bimodal nanoparticle based on the host-guest self-assembly for targeted cellular imaging. *Talanta* 171**,** 8-15. doi:10.1016/j.talanta.2017.04.046

Wang, P., Yoo, B., Sherman, S., Mukherjee, P., Ross, A., Pantazopoulos, P., et al. (2016). Predictive imaging of chemotherapeutic response in a transgenic mouse model of pancreatic cancer. *Int. J. Cancer* 139**,** 712-718. doi:10.1002/ijc.30098

Wang, Q., Yan, H., Jin, Y., Wang, Z., Huang, W., Qiu, J., et al. (2018). A novel plectin/integrin-targeted bispecific molecular probe for magnetic resonance/near-infrared imaging of pancreatic cancer. *Biomaterials* 183**,** 173-184. doi:10.1016/j.biomaterials.2018.08.048

Wang, S., Mao, J., Liu, H., Huang, S., Cai, J., Gui, W., et al. (2020a). pH-Sensitive nanotheranostics for dual-modality imaging guided nanoenzyme catalysis therapy and phototherapy. *J. Mater. Chem. B* 8**,** 4859-4869. doi:10.1039/C9TB02731A

Wang, S., Yin, Y., Song, W., Zhang, Q., Yang, Z., Dong, Z., et al. (2020b). Red-blood-cell-membrane-enveloped magnetic nanoclusters as a biomimetic theranostic nanoplatform for bimodal imaging-guided cancer photothermal therapy. *J. Mater. Chem. B* 8**,** 803-812. doi:10.1039/c9tb01829h

Wang, Z., Ju, Y., Ali, Z., Yin, H., Sheng, F., Lin, J., et al. (2019). Near-infrared light and tumor microenvironment dual responsive size-switchable nanocapsules for multimodal tumor theranostics. *Nat. Commun.* 10**,** 4418. doi:10.1038/s41467-019-12142-4

Wen, R., Lv, X., Yang, T., Li, Y., Tang, Y.A., Bai, X., et al. (2017). Albumin nanoreactor-templated synthesis of Gd_2_O_3_/CuS hybrid nanodots for cancer theranostics. *Sci. China-Mater.* 60**,** 554-562. doi:10.1007/s40843-017-9056-1

Wu, H., Wang, H., Liao, H., Lv, Y., Song, X., Ma, X., et al. (2016a). Multifunctional nanostructures for tumor-targeted molecular imaging and photodynamic therapy. *Adv. Healthcare Mater.* 5**,** 311-318. doi:10.1002/adhm.201500668

Wu, S., Chen, J., Wang, H.C., Chou, M.Y., Chang, T., Yuan, S.S., et al. (2016b). Bispecific antibody conjugated manganese-based magnetic engineered iron oxide for imaging of HER2/neu- and EGFR-expressing tumors. *Theranostics* 6**,** 118-130. doi:10.7150/thno.13069

Xie, M., Zhu, Y., Xu, S., Xu, G., Xiong, R., Sun, X., et al. (2020). A nanoplatform with tumor-targeted aggregation and drug-specific release characteristics for photodynamic/photothermal combined antitumor therapy under near-infrared laser irradiation. *Nanoscale* 12**,** 11497-11509. doi:10.1039/D0NR00123F

Xue, X., Huang, Y., Bo, R., Jia, B., Wu, H., Yuan, Y., et al. (2018a). Trojan horse nanotheranostics with dual transformability and multifunctionality for highly effective cancer treatment. *Nat. Commun.* 9**,** 3653. doi:10.1038/s41467-018-06093-5

Xue, X., Huang, Y., Wang, X., Wang, Z., Carney, R.P., Li, X., et al. (2018b). Self-indicating, fully active pharmaceutical ingredients nanoparticles (FAPIN) for multimodal imaging guided trimodality cancer therapy. *Biomaterials* 161**,** 203-215. doi:10.1016/j.biomaterials.2018.01.044

Yan, R., Hu, Y., Liu, F., Wei, S., Fang, D., Shuhendler, A.J., et al. (2019). Activatable NIR fluorescence/MRI bimodal probes for in vivo imaging by enzyme-mediated fluorogenic reaction and self-assembly. *J. Am. Chem. Soc.* 141**,** 10331-10341. doi:10.1021/jacs.9b03649

Yang, H.M., Park, C.W., Park, S., and Kim, J.D. (2018a). Cross-linked magnetic nanoparticles with a biocompatible amide bond for cancer-targeted dual optical/magnetic resonance imaging. *Colloids Surf. B* 161**,** 183-191. doi:10.1016/j.colsurfb.2017.10.049

Yang, H.Y., Fu, Y., Li, Y., Jang, M.S., Lee, J.H., and Lee, D.S. (2018b). Polymer ligand-assisted fabrication of multifunctional and redox-responsive self-assembled magnetic nanoclusters for bimodal imaging and cancer treatment. *J. Mater. Chem. B* 6**,** 5562-5569. doi:10.1039/c8tb01798k

Yang, H.Y., Jang, M.S., Li, Y., Lee, J.H., and Lee, D.S. (2017). Multifunctional and redox-responsive self-assembled magnetic nanovectors for protein delivery and dual-modal imaging. *ACS Appl. Mater. Interfaces* 9**,** 19184-19192. doi:10.1021/acsami.7b03747

Yang, L., Tang, J., Yin, H., Yang, J., Xu, B., Liu, Y., et al. (2022). Self-assembled nanoparticles for tumor-triggered targeting dual-mode NIRF/MR imaging and photodynamic therapy applications. *ACS Biomater. Sci. Eng.* 8**,** 880-892. doi:10.1021/acsbiomaterials.1c01418

Yang, R., Hou, M., Gao, Y., Lu, S., Zhang, L., Xu, Z., et al. (2019). Biomineralization-inspired crystallization of manganese oxide on silk fibroin nanoparticles for in vivo MR/fluorescence imaging-assisted tri-modal therapy of cancer. *Theranostics* 9**,** 6314-6333. doi:10.7150/thno.36252

Zhang, Q., Wang, W., Zhang, M., Wu, F., Zheng, T., Sheng, B., et al. (2020). A theranostic nanocomposite with integrated black phosphorus nanosheet, Fe_3_O_4_@MnO_2_-doped upconversion nanoparticles and chlorin for simultaneous multimodal imaging, highly efficient photodynamic and photothermal therapy. *Chem. Eng. J.* 391**,** 123525. doi:10.1016/j.cej.2019.123525

Zhang, X., Li, X., Sun, S., Wang, P., Ma, X., Hou, R., et al. (2021). Anti-tumor metastasis via platelet inhibitor combined with photothermal therapy under activatable fluorescence/magnetic resonance bimodal imaging guidance. *ACS Appl. Mater. Interfaces* 13**,** 19679-19694. doi:10.1021/acsami.1c02302

Zhao, H., Richardson, R., Talebloo, N., Mukherjee, P., Wang, P., and Moore, A. (2019). uMUC1-Targeting magnetic resonance imaging of therapeutic response in an orthotropic mouse model of colon cancer. *Mol. Imaging Biol.* 21**,** 852-860. doi:10.1007/s11307-019-01326-5
